# The Nuclear Protein Sge1 of *Fusarium oxysporum* Is Required for Parasitic Growth

**DOI:** 10.1371/journal.ppat.1000637

**Published:** 2009-10-23

**Authors:** Caroline B. Michielse, Ringo van Wijk, Linda Reijnen, Erik M. M. Manders, Sonja Boas, Chantal Olivain, Claude Alabouvette, Martijn Rep

**Affiliations:** 1 Plant Pathology, Swammerdam Institute for Life Sciences, University of Amsterdam, Amsterdam, The Netherlands; 2 UMR 1229 INRA Université de Bourgogne Microbiologie du Sol et de l'Environnement, Dijon, France; University of Melbourne, Australia

## Abstract

Dimorphism or morphogenic conversion is exploited by several pathogenic fungi and is required for tissue invasion and/or survival in the host. We have identified a homolog of a master regulator of this morphological switch in the plant pathogenic fungus *Fusarium oxysporum* f. sp. *lycopersici*. This non-dimorphic fungus causes vascular wilt disease in tomato by penetrating the plant roots and colonizing the vascular tissue. Gene knock-out and complementation studies established that the gene for this putative regulator, *SGE1* (*SIX* Gene Expression 1), is essential for pathogenicity. In addition, microscopic analysis using fluorescent proteins revealed that Sge1 is localized in the nucleus, is not required for root colonization and penetration, but is required for parasitic growth. Furthermore, Sge1 is required for expression of genes encoding effectors that are secreted during infection. We propose that Sge1 is required in *F. oxysporum* and other non-dimorphic (plant) pathogenic fungi for parasitic growth.

## Author Summary

Plant pathogenic fungi have evolved many ways to infect their hosts and can have devastating effects on commercial crop production. Dissecting their infection strategies and understanding the molecular pathways involved in pathogenesis have been and continue to be the subject of intensive research. New insights gained may help to develop disease controlling strategies. *Fusarium oxysporum* has become a model for root invading, pathogenic fungi. This fungus attacks a wide range of plant species worldwide and the only effective disease control strategies in the field are crop rotation and usage of resistant plant varieties, if at all available. In the last decade, many genes have been identified that play a role during pathogenesis, many of which are linked to general strain fitness. Only few genes have been identified that are required for pathogenesis but do not affect vegetative growth. This paper describes the characterization of such a gene. Its protein product, Sge1, is conserved in the fungal kingdom and represents a new class of transcriptional regulators involved in morphological switching in dimorphic fungal pathogens. In *F. oxysporum*, the Sge1 protein is required for parasitic growth and is associated with expression of parasitic phase-specific genes. We suggest that the function of Sge1 is conserved in (plant) pathogenic fungi.

## Introduction

The fungus *Fusarium oxysporum* is found in both agricultural and non-cultivated soils throughout the world. The species consists of non-pathogenic and pathogenic isolates, both known as efficient colonizers of the root rhizosphere. The pathogenic isolates, grouped into *formae specialis* depending on their host range [1],[2], cause wilt or rot disease in important agricultural and ornamental plant species, such as tomato, banana, cotton and tulip bulbs, thereby causing serious problems in commercial crop production [3],[4]. Recently, *F. oxysporum* has also been reported as an emerging human pathogen, causing opportunistic mycoses [5]–[7].

In the absence of plant roots *F. oxysporum* survives in the soil either as dormant propagules (chlamydospores) or by growing saprophytically on organic matter [1],[8]. When growing on roots of a suitable host *F. oxysporum* appears to switch from a saprophyte into a pathogen. As a pathogen *F. oxysporum* needs to overcome host defence responses and sustain growth within the host in order to establish disease. To do so, *F. oxysporum* likely undergoes reprogramming of gene expression. In the last decade, genes have been identified that do not seem to be required for saprophytic growth, but are involved in or required for pathogenicity and/or are specifically expressed during *in planta* growth. Examples are *SIX1*, encoding a small secreted protein, and *FOW2* and *FTF1*, both encoding Zn(II)_2_Cys6-type transcriptional regulators [9]–[12].

In an insertional mutagenesis screen aimed at identification of pathogenicity factors of *Fusarium oxysporum* f. sp. *lycopercisi* (*Fol*), a gene now called *SGE1* (*SIX* Gene Expression 1) was identified that shows homology to the transcriptional regulators *Candida albicans WOR1* and *Histoplasma capsulatum RYP1* [9]. These transcription factors have been identified as major regulators of morphological switching in these human pathogens: from a filamentous to a yeast form in *H. capsulatum* and from a white to opaque cell type in *C. albicans* [10]–[13]. In both fungi, these morphological transitions are correlated with the ability to cause disease. Targeted deletion of *RYP1* in *H. capsulatum* or *WOR1* in *C. albicans* locks the fungus in its filamentous form or white cell type, respectively.

In this work we characterize *SGE1* and show that it shares many characteristics with *WOR1* and *RYP1*. In addition, we show that expression of effector genes is lost in the *SGE1* deletion mutant. We conclude that Sge1 plays a major role during parasitic growth, defined as extensive *in planta* growth leading to wilt symptoms, in *F. oxysporum* f. sp. *lycopersici*.

## Results

### Isolation and characterization of *SGE1* and *FoPAC2*

**Figure 1:**
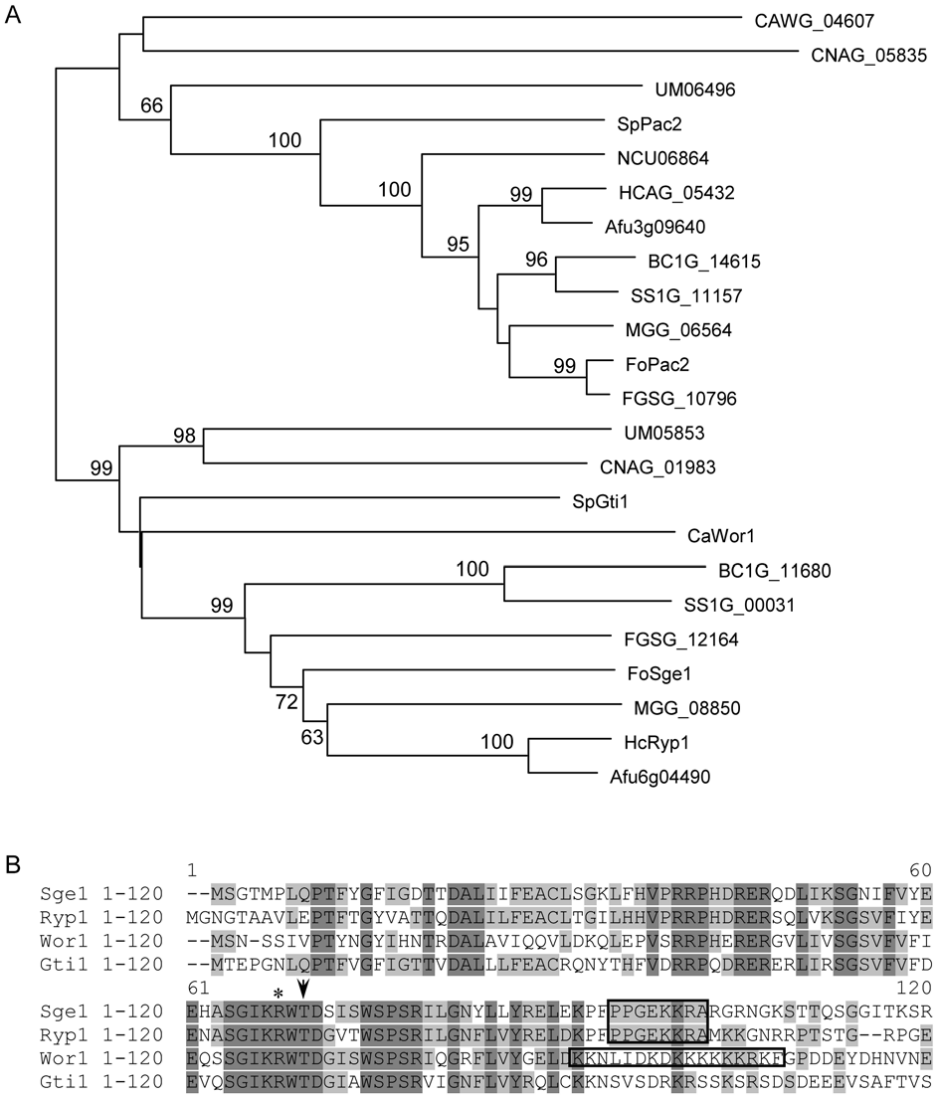
Representation of Sge1 homologs in fungi. A) Phylogenetic (neighbour joining, mid-point rooted) tree of Sge1 and FoPac2 with homologs from *C. albicans* (Wor1 and CAWG_04607), *S. pombe* (SpGti1 and SpPac2), *H. capsulatum* (HcRyp1 and HCAG_05432), *Magnaporthe grisea* (MGG_08850 and MGG_06564), *Aspergillus fumigatus* (Afu6g04490 and Afu3g09640), *Fusarium graminearum* (FGSG_12164 and FGSG_10796), *Botrytis cinerea* (BC1G_11680 and BC1G_14615), *Sclerotinia sclerotiorum* (SS1G_00031 and SS1G_11157), *Cryptococcus neoformans* (CNAG_01983 and CNAG_05835), *Ustilago maydis* (UM05853 and UM06496) and *Neurospora crassa* (NCU06864), constructed using MacVector software. Only fully aligned parts of the multiple sequence alignment were used (manual curation). Bootstrap percentages are provided only for branches receiving 60% or more support (1000 replications). Branch length reflects the extent of sequence divergence. B) Protein sequence alignment of the Sge1 N-terminal region with *H. capsulatum* Ryp1, *C. albicans* Wor1 and *S. pombe* Gti1. Conserved residues are shaded black, similar residues are shaded grey. The arrow head indicates the conserved threonine residue within the potential protein kinase A phosphorylation site and the asterisk indicates the mutated residue in Sge1R66S. Predicted nucleur localization signals are boxed. The protein sequence alignment were created using VectorNTI software.

In an insertional mutagenesis screen aimed at identifying genes involved in pathogenicity a non-pathogenic mutant (5G2) and one severely reduced in pathogenicity (101E1) were identified that both carried a single T-DNA insertion into the ORF of *FOXG_10510* [9], hereafter called *SGE1* (*SIX* Gene Expression 1). The *SGE1* ORF contains no introns and encodes a protein of 330 amino acids (http://www.broad.mit.edu/annotation/genome/fusarium_group/MultiHome.html). Sequence analysis revealed that the N-terminus (amino acids 1–120) contains a TOS9 (COG5037) and a Gti1_Pac2 family domain (Pfam09729) and is conserved in the fungal kingdom; all fungi of which the genome sequence was examined, including ascomycetes, basidiomycetes and zygomycetes, contain related genes that divide in two groups based on sequence similarity of the predicted proteins. Most ascomycetes have one member in each group, except *Neurospora crassa* which lacks a member of the *SGE1* group (Figure 1A). The basidiomycete *Coprinus cinereus* and the zygomycete *Rhizopus oryzae* contain more than two members, still with at least one member in each group (data not shown). The branching order within the two groups does not always follow species phylogeny, making orthology questionable. Examples are the placement of FoSge1 and FGSG_12164 basal to the *Magnaporthe grisea* homolog (MGG_00850), the placement of CAWG_04607 of *C. albicans* basal to Pac2 of *Schizosaccharomyces pombe* (and closer to basidiomycete homologs) and of NCU06864 of *N. crassa* basal to homologs of other filamentous fungi (pezizomycotina) (Figure 1A). Sge1 is in the same group as *Histoplasma capsulatum* Ryp1 and *Candida albicans* Wor1, both identified as regulators for morphological switching [10]–[13], and *Schizosaccharomyces pombe* Gti1, which plays a role in gluconate uptake upon glucose starvation [14]. A potential protein kinase A phosphorylation site (KRWTDS/G) is conserved between these proteins (Figure 1B). In addition, a nuclear localization motif is present in Sge1 (+93 to +100) that is shared with Ryp1 (Figure 1B). The *F. oxysporum* protein related to Sge1, encoded by *FOXG_12728*, shows high similarity to *S. pombe* Pac2, a protein controlling the onset of sexual development [15]. Interestingly, in the same insertional mutagenesis screen mentioned above, a *Fol* mutant with reduced pathogenicity (30C11) was identified in which one of two T-DNA insertions resides in the *FOXG_12728* ORF [9], hereafter called *FoPAC2*.

### *SGE1* is strictly required for pathogenicity

**Figure 2:**
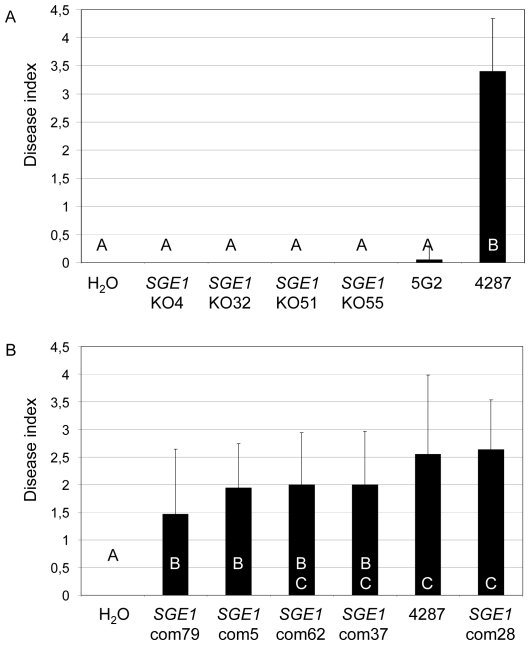
Sge1 is required for full pathogenicity. Nine to eleven days old tomato seedlings were inoculated with fungal spore suspensions using root-dip inoculation and the disease index (ranging from 0, healthy plant to 4, severely diseased plant or dead plant) was scored after three weeks. Error bars indicate standard deviation and capitals define statistically different groups (ANOVA, p = 0.95). A) Average disease index of 20 plants three weeks after mock inoculation (H_2_O) or inoculation with four independent *SGE1* deletion mutants (*SGE1*KO4, 32, 51, and 55), insertional mutant 5G2 or wild type (4287). B) Average disease index of 20 plants three weeks after mock inoculation (H_2_0) or inoculated with five independent *SGE1* complementation strains (*SGE1*com5, 37, 62, and 79) or wild type (4287).

**Figure 3:**
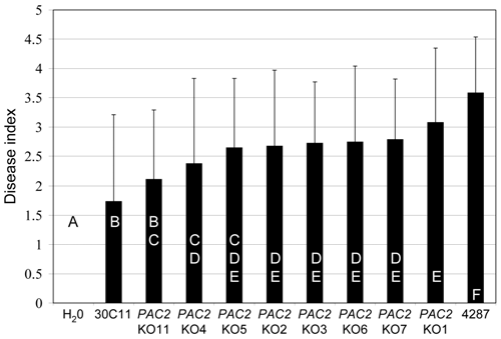
FoPac2 only plays a minor role in pathogenicity. Nine to eleven days old tomato seedlings were inoculated with fungal spore suspensions using root-dip inoculation [50] and the disease index (ranging from 0, healthy plant to 4, severely diseased plant or dead plant) was scored after three weeks. Error bars indicate standard deviation and capitals define statistically different groups (ANOVA, p = 0.95). Average disease index of 20 plants three weeks after mock inoculation (H_2_O) or inoculation with eight independent *FoPac2* deletion mutants (*FoPac2*KO1, 2, 3, 4, 5, 6, 7, and 11), insertional mutant 30C11 or wild type (4287).

To assess the involvement of *SGE1* and *FoPAC2* in pathogenicity, gene knock-out mutants were generated by homologous recombination. Four independent *SGE1* and eight independent *FoPAC2* knock-out mutants were obtained, with the deletions confirmed by PCR and Southern analysis (Figure S1, S3 and S4). The *SGE1* deletion mutants were non-pathogenic on tomato in a root dip bioassay and corroborated the severely reduced to non-pathogenic phenotype of the insertional mutagenesis mutants (Figure 2A). Re-introduction of the *SGE1* gene *in locus* by homologous recombination in a knock-out mutant (Figure S2 and S3) restored pathogenicity (Figure 2B), confirming that the loss of pathogenicity was due to loss of *SGE1*. Deletion of *FoPAC2* only had a minor effect on pathogenicity. All knock-out mutants were significantly different from the wild type in disease causing ability, but seven out of the eight mutants were also significantly different from the original insertion mutant (30C11) (Figure 3). This indicates that *FoPAC2* plays at most a minor role during infection and that most probably additional defects in the 30C11 mutant added to the reduced pathogenicity phenotype. Since the loss of pathogenicity was complete upon deletion of *SGE1*, we focussed on this gene for further analysis.

### *SGE1* is not required for vegetative growth, but quantitatively affects conidiation

**Figure 4:**
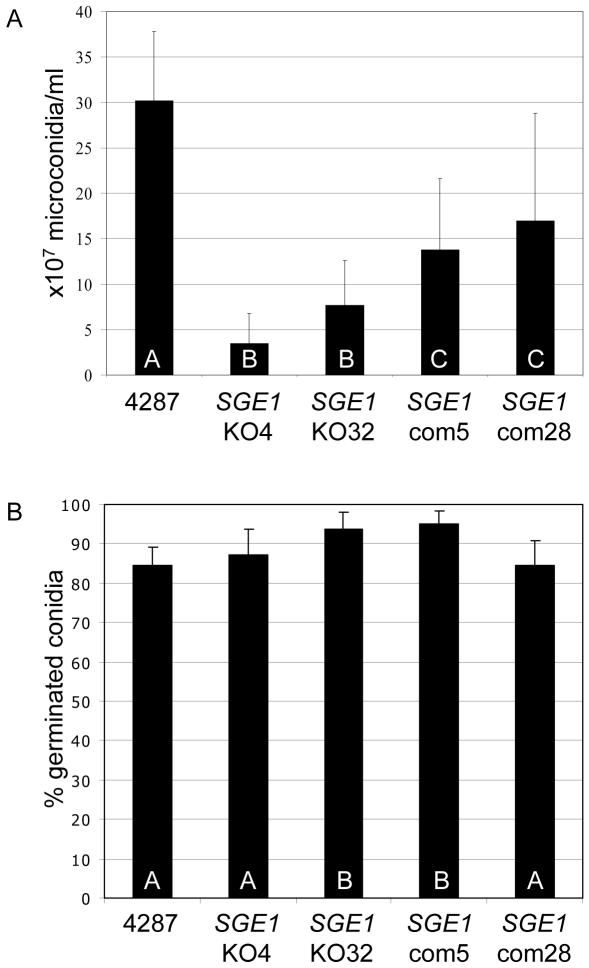
Sge1 is involved in conidiogenesis. Microconidia production was determined after five days of growth in minimal medium and counted in a Bürker-Türk haemocytometer. A) The average number of microconidia of five independent experiments each with five replicates. B) The average germination rate of the isolated microconidia on PDA medium after six hours of incubation at 25°C. Error bars indicate standard deviation and capitals define statistically different groups (ANOVA, p = 0.95).

Previously, we reported that vegetative growth of the insertional mutants 5G2 and 101E1 are indistinguishable from that of the wild type on various carbon sources [9]. To more fully analyze potential metabolic defects of the *sge1* mutant, we made use of BIOLOG FF MicroPlates, in which each well contains a different carbon source [16]. Also in this assay no reproducible differences were observed between growth of the wild type and the *SGE1* deletion mutant on 95 different carbon sources (Figure S5). Microconidia and macroconidia generated in minimal or CMC liquid medium were phenotypically indistinguishable from wild type (Figure S6). However, the *SGE1* deletion mutants produced about 6-fold less microconidia compared to the wild type in both media, and this phenotype was only partially restored in the *SGE1* complementation mutants (Figure 4A). The conidial germination rates were comparable to wild type (Figure 4B), indicating that, although less microconidia are formed, they are fully viable. These observations indicate that *SGE1* is quantitatively involved in conidiogenesis, but is not required for conidial fitness, overall (colony) morphology, vegetative growth or carbon source utilization.

### *SGE1* expression is upregulated during infection of tomato roots

**Figure 5:**
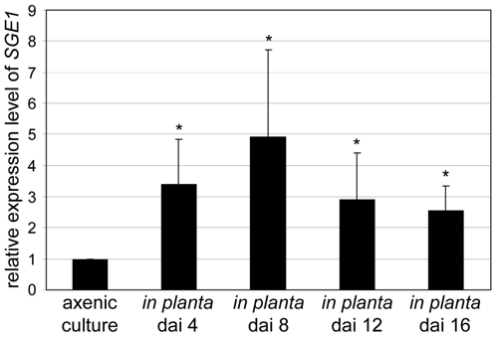
*SGE1* is upregulated during infection of tomato roots. Nine to eleven days old tomato seedlings were inoculated with either water or wild type *Fol* using root-dip inoculation. Roots were harvested 4, 8, 12, and 16 days after inoculation followed by RNA isolation and cDNA synthesis. Quantitative PCR was used to determine the expression levels of the constitutively expressed gene *EF-1α* and *SGE1* in axenic culture (minimal medium) and during infection. Relative expression levels were subsequently calculated using the comparative C(t) method. *, significantly different from the *SGE1* expression level in axenic culture at a 95% confidence interval.

*C. albicans WOR1* and *H. capsulatum RYP1* are 45-fold and 4-fold upregulated upon transition from white to opaque cells in *C. albicans* and from filamentous growth to yeast cells in *H. capsulatum*, respectively [11],[17]. To determine the relative expression levels of *SGE1* during saprophytic and parasitic growth quantitative PCR was performed. Expression levels of *SGE1* were determined both in axenic culture and during tomato root infection at different time points after inoculation and compared to the level of the constitutively expressed elongation factor 1 alpha gene (*EF-1α*). We found that *SGE1* expression is upregulated 2- to 5-fold during infection with maximal expression eight days after inoculation (Figure 5).

### *SGE1* is not essential for colonization or penetration of the root surface

**Figure 6:**
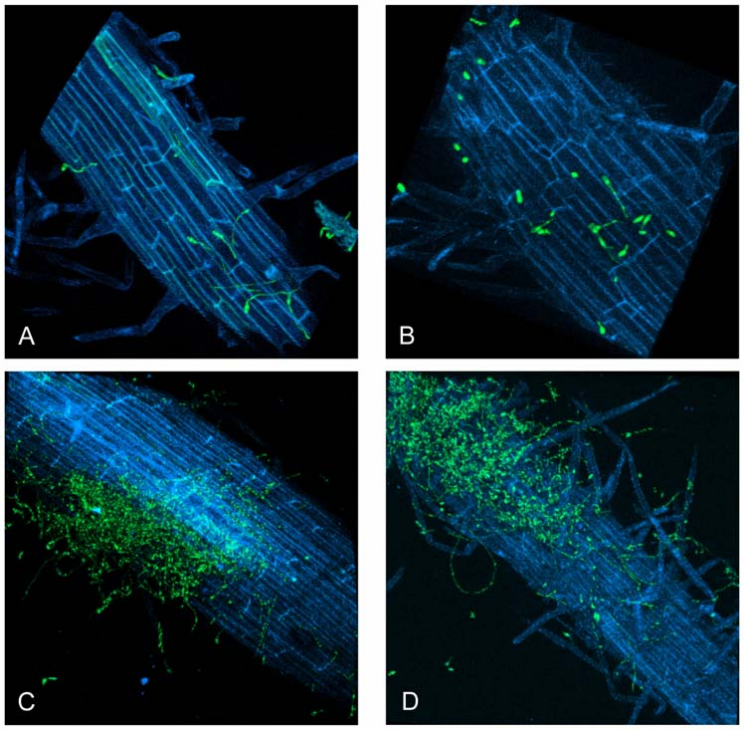
The *SGE1* deletion mutant is not impaired in root colonization. The infection behaviour of the *SGE1* deletion mutant was determined by confocal microscopy. Nine to eleven days old tomato seedlings were inoculated with *SGE1* deletion mutant spore suspension and root colonization was determined after one to two days after inoculation. Germinated spores were observed on a tomato root infected with a GFP-expressing virulent strain (A) or with the *SGE1* deletion mutant (B). Patches of colonization were found on a tomato root infected with a GFP-expressing virulent strain (C) or with the *SGE1* deletion mutant (D).

**Figure 7:**
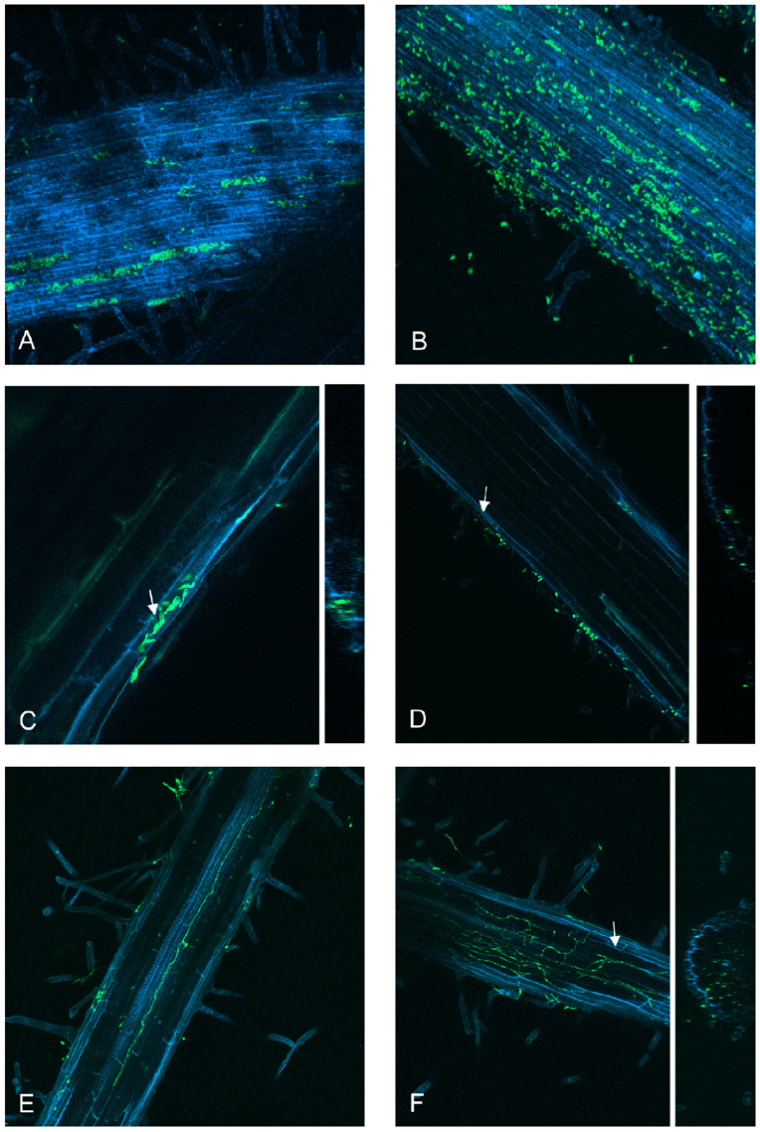
The *SGE1* deletion mutant is not impaired in penetration. The infection behaviour of the *SGE1* deletion mutant was determined by confocal microscopy. Nine to eleven days old tomato seedlings were inoculated with *SGE1* deletion mutant spore suspension and root colonization was determined after three to four days after inoculation. Fungal growth within single cell compartments was observed in a tomato root infected with a GFP-expressing virulent strain (A). More dispersed fungal growth was observed in a tomato root infected with the *SGE1* deletion mutant (B). Fungal growth within cells was observed in a tomato root infected with a GFP-expressing virulent strain (C) or, less frequently, with the *SGE1* deletion mutant (D). Fungal growth within the xylem vessel of a tomato root was seen upon infection with a GFP-expressing virulent strain (E) and intercellular growth was seen upon infection with the *SGE1* deletion mutant (F). The arrows indicate the position in the YZ projection.

To determine at which stage during infection the *SGE1* deletion mutant is halted, tomato root colonization by the mutant was visualized using fluorescent binocular and confocal laser scanning microscopy. Tomato seedlings were infected with a GFP expressing wild type strain or *SGE1* deletion strain and colonization was followed over time. After two days, patches of colonization on the roots were observed for both strains (Figure S7), indicating that the *SGE1* deletion mutant is not impaired in root surface colonization. This observation was confirmed by confocal laser scanning microscopy. For both strains, germinated spores were observed on the root surface 24 hours after infection (Figure 6A and 6B) and mycelial mass increased in time leading to patches of colonization as described above (Figure 6C and 6D). No difference in colonization was observed between the wild type and the *sge1* mutant in the first 2 days after inoculation. However, after three days, plant cells filled with spores and mycelium were observed in roots infected with the wild type strain (Figure 7A), indicating that penetration of the root surface had occurred (Figure 7C). A mixture of germinated spores and mycelium was also observed on plant roots infected with the *sge1* mutant, however, growth was dispersed over the entire root (Figure 7B) and was not confined to single plant cells as observed for the wild type. Occasionally, the *sge1* mutant was observed within the root cortex (Figure 7D), indicating that the *sge1* mutant is capable of penetrating the surface. This is in line with our observation that the *sge1* mutant is able to penetrate a cellophane sheet (Figure S8), an assay used to assess the ability to form penetration hyphae [18]. Four days after infection fungal growth within the roots was observed. In roots infected with the wild type, growth within the xylem vessel was observed once (Figure 7E). Also in one case, extensive growth of the *sge1* mutant was observed within the root (Figure 7F); however, this growth was mainly intercellular. In both cases the fungal growth extended from a point where the root was broken. Since these cases were too rare for a proper quantification, we can only conclude that both the wild type and the *sge1* mutant are capable of *in planta* growth.

To determine whether the *sge1* mutant is able to colonize xylem vessels as extensively as the wild type, tomato seedlings were inoculated with the wild type, *SGE1* knock-out or the *SGE1* complementation strain and potted according to the bioassay procedure (i.e. with damaged roots to allow relatively easy entry into vessels). One week after inoculation the hypocotyl was cut in slices of several millimeters and put on rich (PDA) medium. After two days *F. oxysporum* outgrowth was observed from hypocotyl pieces previously inoculated with the wild type and the *SGE1* complementation strain, but not from the hypocotyl pieces previously inoculated with the *SGE1* knock-out mutant (Figure S9). Based on the above described observations, it can be concluded that Sge1 is not required for early pathogenesis-related functions, such as root colonization and penetration, but appears to be required for extensive growth within plant cells and the xylem.

### Sge1 is localized in the nucleus

**Figure 8:**
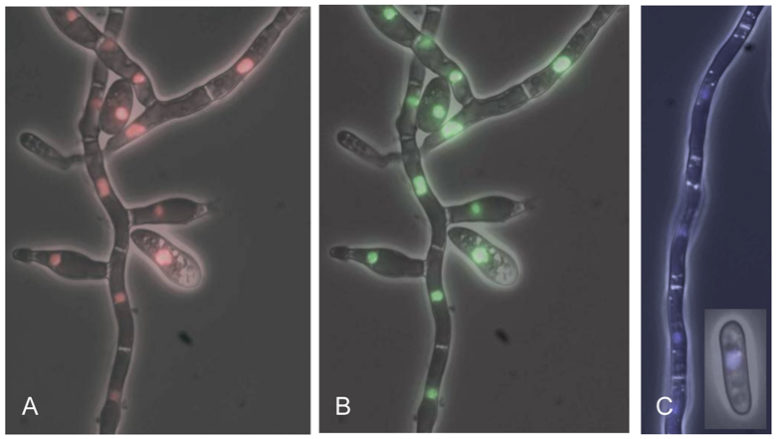
Sge1 is localized in the nucleus. The subcellular localization of the fluorescent proteins Sge1::RFP and Sge1::CFP was determined after five days of growth in minimal medium using a fluorescence microscope. A) Subcellular localization of Sge1::RFP. B) Subcellular localization of H2B::GFP expressed in the Sge1::RFP background. C) Subcellular localization of Sge1::CFP.

The homologs of Sge1 in *C. albicans* and *H. capsulatum* are nuclear proteins [10]–[13]. To determine whether this is also the case for Sge1 its subcellular localization was investigated. For this purpose, Sge1 was fused C-terminally to the fluorescent proteins CFP or RFP. Constructs expressing these fusion proteins were introduced in the *SGE1* deletion mutant by homologous recombination at the *sge1* locus (verified by Southern analysis (Figure S3)). The functionality of the fusion proteins was determined in a bioassay. *SGE1::CFP* restored pathogenicity to almost wild type levels (Figure S10). Disease symptoms were also observed upon infection with strains expressing *SGE1::RFP*, albeit severely reduced compared to the wild type infection (Figure S10). Subcellular localization for both fusion proteins was similar in that they are localized in the nucleus in both spores and hyphae (Figure 8). Nuclear localization was verified by introduction of a construct encoding histone H2B::GFP [19] into the *SGE1::RFP* strain. Both fusion proteins localized to the same compartment (Figure 8). These observations support the potential role of Sge1 as a transcriptional regulator in *F. oxysporum*.

### The putative Pka phosphorylation site is required for Sge1 function

**Figure 9:**
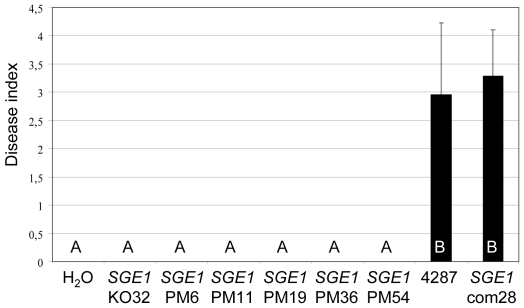
An intact Pka phosphorylation site in Sge1 is essential for pathogenicity. Nine to eleven days old tomato seedlings were inoculated with fungal spore suspensions using root-dip inoculation and the disease index (ranging from 0, healthy plant to 4, severely diseased plant or dead plant) was scored after three weeks. Error bars indicate standard deviation and capitals define statistically different groups (ANOVA, p = 0.95). Average disease index of 20 plants three weeks after mock inoculation (H_2_O) or inoculation with five independent Sge1R66S complementation mutants (*SGE*PM6, 11, 19, 36 and 54), an *SGE1* complementation mutant (*SGE1*com28) or wild type (4287).

As described above, the Sge1 protein contains a potential Pka phosphorylation site and this site is conserved in all Sge1 homologs (Figure 1B). Replacement of the conserved threonine residue by an alanine impaired *S. pombe* Gti1 function [14]. In an attempt to identify Sge1 interacting partners using a yeast two-hybrid screen, the *SGE1* gene, fused to the portion of *GAL4* encoding the Gal4 DNA binding domain, was introduced in *Saccharamyces cerevisiae*. To our surprise, after numerous transformation attempts only one colony was obtained. After re-isolation of the plasmid from this colony followed by sequencing it turned out that a point mutation in the *SGE1* sequence (C196A) had occurred resulting in an amino acid change from an arginine into a serine at position 66, altering the Pka phosphorylation site (Figure 1). In contrast to wild type *SGE1*, this mutant form of *SGE1* could be easily re-transformed to yeast. We concluded that *SGE1* is normally toxic in *S. cerevisiae* and that the R>S mutation leads to tolerance in this yeast. To determine the effect of this mutation on the activity of Sge1 in *Fol*, a construct expressing Sge1R66S was introduced in the *SGE1* deletion mutant. Correct gene replacement was again confirmed by PCR (Figure S11). All strains encoding the Sge1R66S protein were non-pathogenic (Figure 9), suggesting that an intact Pka phosphorylation site is required for Sge1 to function properly.

### *SGE1* regulates expression of effector genes

**Figure 10:**
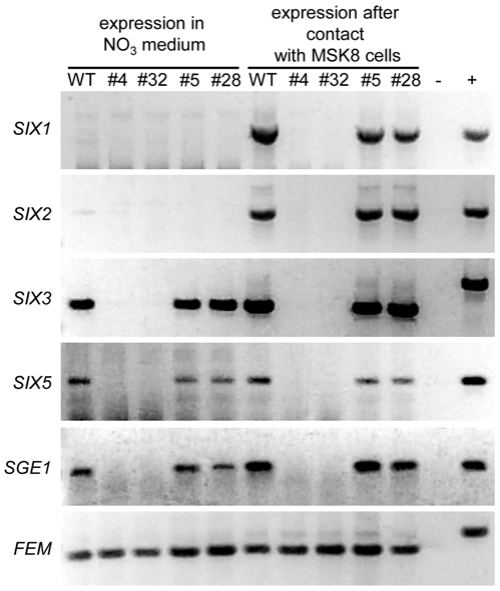
*SGE1* regulates expression of effector genes. Expression levels of *SIX1*, *SIX2*, *SIX3*, *SIX5*, *SGE1* and the constitutively expressed gene *FEM1* were determined in the *SGE1* deletion (#4 and #32) and complementation mutants (#5 and #28) and in the wild type (WT) grown *in vitro* and under *in planta*-mimicking conditions. Total RNA was isolated from mycelium harvested after five days of growth in minimal medium (*in vitro*) and from mycelium grown for 24 h in BY-medium in the presence of one week old MSK8 cells (*in planta*-mimicking) followed by RT-PCR. −, negative control. +, positive (genomic DNA) control.

The transcriptional regulators Ryp1 and Wor1 regulate expression of phase specific genes [11],[20]. Since Sge1 shares many features with Ryp1 and Wor1, we hypothesized that expression of *F. oxysporum* genes specifically expressed during infection could be altered. Examples of such genes are those encoding effectors, small *in planta* secreted proteins, called Six (Secreted in xylem) proteins in *Fol*. Recently, it was shown that *F. oxysporum* secretes numerous Six proteins during infection [21] (Houterman and Rep, unpublished). *SIX1*, encoding Avr3, is strongly induced upon penetration of the root cortex and plays a role during pathogenicity [22],[23]. In addition, Six3 (Avr2) and Six4 (Avr1) have been shown to play a role in virulence as well as resistance protein recognition [24],[25]. Since the *SGE1* deletion mutant does not show extensive *in planta* growth, precluding assessment of *SIX* gene expression during root infection, we incubated the mutant together with tomato cells in culture, a situation known to induce *SIX1* expression [22]. MSK8 tomato cells were inoculated with a conidial suspension of the wild type or the *SGE1* deletion mutant. After 24 h the cultures were harvested and the presence of *SIX* gene transcripts was determined by RT-PCR and compared to transcript levels in axenic cultures. As expected, *SIX1* gene expression was very low when the wild type strain was grown in axenic culture and expression was strongly upregulated upon incubation with MSK8 cells. A similar expression pattern was observed for *SIX2* (Figure 10). In contrast, the *SIX3* and *SIX5* genes were expressed in the wild type strain in axenic culture as well as in the presence of MSK8 cells (Figure 10). Interestingly, in the *sge1* mutant expression of all four *SIX* genes was lost both in axenic culture and in the presence of MSK8 cells. Expression was restored in the complemented strain (Figure 10). These results show that expression of at least four *SIX* genes tested is dependent on the presence of *SGE1*.

### The *SGE1* deletion mutant exhibits biocontrol activity

**Figure 11:**
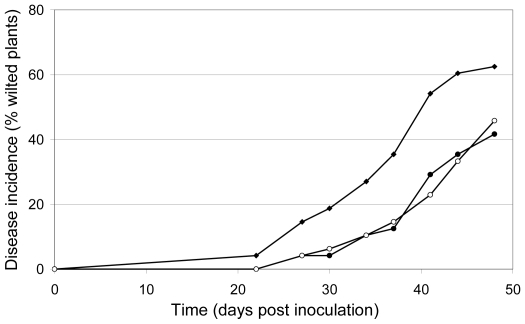
The *sge1* mutant and its parental strain exhibit biocontrol activity on flax. The protective capacity of the *sge1* mutant and its parental strain were determined on flax. Flax cv. viking was inoculated with the pathogenic isolate *F. oxysporum* f. sp. *lini* (10^4^ conidia/ml) alone or in combination with the *sge1* mutant or the 4287 wild type strain (10^6^ conidia/ml). Fusarium wilt incidence is expressed as percentage of wilted plants. Black diamond, *F. oxysporum* f. sp. *lini*. Open circle, *F. oxysporum* f. sp. *lini* and *sge1* mutant. Black circle, *F. oxysporum* f. sp. *lini* and the wild type strain 4287.

Biocontrol activity has been reported to be dependent on colonization of superficial cell layers, a capacity shared between pathogenic and protective strains and has been reported to be independent of competition for putative penetration sites or nutrients [26]. *Fusarium oxysporum* f. sp. *lycopersici* exerts biocontrol activity on flax when added in a 100-fold excess relative to a *F. oxysporum* strain pathogenic towards flax. To determine whether the *sge1* mutant is still able to protect flax against wilt disease like its parental strain, flax cv. viking was treated with either a pathogenic isolate of *F. oxysporum* f. sp. *lini* (Foln3) alone or in a 1∶100 ratio with either the *sge1* mutant or the wild type strain 4287. The first wilt symptoms were observed 22 days after inoculation in the treatment with Foln3 (Figure 11). In the Foln3/*sge1* mutant and Foln3/wild type combination treatments the first disease symptoms were delayed by 3 days. Disease symptoms were always reduced in the Foln3/*sge1* mutant and Foln3/wild type treatments compared to the Foln3 treatment: 48 days post inoculation disease symptoms were reduced by 27 and 33%, by the *sge1* mutant and the wild type, respectively (Figure 11). The ANOVA performed on AUDPCs indicated that this difference was significant at the probability of 91%. We conclude that the *sge1* mutant is able to protect flax against Fusarium wilt as well as the wild type strain, suggesting that it can colonize roots efficiently.

## Discussion

Fungi have various ways to adapt their morphology to the environment, one example being dimorphism. Dimorphism is a strategy frequently employed by fungal pathogens where this developmental transition is correlated with the ability to cause disease (reviewed by [27],[28]). In this study, we have characterized Sge1, the *F. oxysporum* homolog of the master regulator of morphological switching Wor1 and Ryp1. Although *F. oxysporum* does not display an immediately obvious morphological switch like *C. albicans* or *H. capsulatum*, Sge1 was found to be required for colonization of the xylem system and disease development.

Sge1, together with its homolog FoPac2, described in this work, belong to a class of conserved fungal proteins. Both proteins have apparent orthologs across the fungal kingdom and the N-terminal domain of these proteins is always more conserved than the C-terminus, which is generally rich in glutamine residues. Despite their similarity, the *SGE1* and *FoPAC2* deletion mutants generated in this study display different phenotypes, indicating that the functions of these proteins are not redundant. The FoPac2 and Sge1 homologs in *S. pombe*, Pac2 and Gti1, respectively, also have a different function although both proteins are involved in regulation of transition processes. Pac2 is a negative regulator of sexual development, which is induced under nitrogen starvation [15] and Gti1 is a positive regulator of metabolic reprogramming by inducing gluconate uptake under glucose starvation [14]. The best characterized members of the class to which Sge1 belongs are Wor1 in *C. albicans* and Ryp1 in *H. capsulatum* [10]–[13].

Ryp1 and Sge1 share a conserved and apparently functional nuclear localization signal. In addition, all three proteins contain a putative protein kinase A (Pka) phosphorylation site. Another common feature of these proteins is increased expression of their genes upon transition. *WOR1* and *RYP1* are both under tight transcriptional regulation in that hardly any expression of these genes can be detected in the white and the filamentous growth phase, respectively. Their expression is upregulated 45- and 4-fold during and after the transition to the opaque or the yeast growth phase [10],[11],[13],[17]. Although *SGE1* expression can easily be detected in the saprophytic growth phase, it was found to be upregulated 2- to 5-fold during *in planta* growth. *WOR1* is preceded by an exceptionally large intergenic region of 10.3 kb (average length in *C. albicans* ∼0.9 kb (http://www.broad.mit.edu/annotation/genome/candida_albicans/GeneStatsSummary.html) and it is able to bind to several positions in its own upstream region, although the protein does not contain an annotated DNA binding domain [13]. Also Ryp1, which is preceded by an intergenic region of about 2 kb, is able to bind to several positions in its own upstream region [11]. The intergenic region preceding *SGE1* is larger than the average in the *F. oxysporum* genome: 4.3 kb versus 2.0 kb (http://www.broad.mit.edu/annotation/genome/fusarium_group/GeneStatsSummary.html), but whether Sge1 is able to activate its own transcription by binding to its promoter remains to be elucidated. Nevertheless, given the nuclear localization of Sge1, the homology to the transcriptional regulators Wor1 and Ryp1, and the effect of deletion of *SGE1* on expression of *SIX* genes, we propose that also Sge1 also acts as a transcriptional regulator.

The requirement of Sge1 for expression of effector genes may explain the non-pathogenic phenotype of the *SGE1* deletion mutant. Based on the apparent absence of xylem colonization as observed with confocal microscopy and the absence of the *sge1* mutant in hypocotyls from infected plants, we conclude that although the mutant is still able to colonize roots and penetrate the root surface, it is not able to grow extensively in living cells or the xylem system. The ability of the *sge1* mutant to colonize roots superficially is supported by the biocontrol activity of the *sge1* mutant. The only additional phenotype of the mutant that we found was a 6-fold lower microconidia production compared to the wild type strain. An aberrant conidiogenesis behaviour was also observed for the *H. capsulatum ryp1* mutant [11], indicating a conserved role for these proteins in conidiogenesis. In *C. albicans*, the regulatory network that drives white-opaque switching, to which Wor1 belongs, is beginning to be understood. A gene counteracting *WOR1* is *EFG1*, which is necessary for maintaining the white state [29]. Wor1 is able to bind to the upstream region of *EFG1*, thereby regulating its expression [20]. Interestingly, the *Fusarium* homolog of Efg1 is FoStuA, a protein required for conidiogenesis in *F. oxysporum* [30]. However, *STUA* expression levels in the wild type and in the *SGE1* deletion mutant were comparable (data not shown). How Sge1 affects conidiogenesis remains, therefore, to be elucidated.

As mentioned above, a common feature of Sge1 and its homologs is the presence of a potential protein kinase A phosphorylation site. The functionality of this phosphorylation site has been investigated in *S. pombe*. It was shown that gluconate import under repressing conditions (high glucose concentration) occurred in a protein kinase A deletion mutant. Therefore, it was speculated that Pka1 inhibits Gti1 protein function. However, alteration of the Pka phosphorylation site by replacement of the conserved threonine residue by an alanine did not result in activation of the Gti1 protein and the expected gluconate import was not observed, indicating that either Pka1 does not inhibit Gti1 activity by phosphorylation or that the mutation impairs Gti1 function or its stability [14]. In Sge1, the same Pka site appears to be required for activity. A stable transformant of *SGE1* in *S. cerevisiae* was obtained only after a spontaneous mutation of an arginine into a serine in the potential phosphorylation site, suggesting that expression of wild type *SGE1* in yeast inhibits growth. *S. cerevisiae* contains a *SGE1* homolog, *YEL007W*, and this gene has been implicated to play a role in regulation of smooth ER, cell adhesion and budding [31]–[33]. It could be that expression of both genes is lethal to yeast. Introduction of the mutated *SGE1* gene in the *SGE1* deletion mutant of *Fol* failed to restore the pathogenicity defect, confirming that the R66S mutation impairs Sge1 function, possibly due to an effect on phosphorylation. Since there is only a minor increase in *SGE1* expression levels upon *in planta* growth, post-translational modifications such as phosphorylation could play a key role in activation of Sge1. Unfortunately, initial attempts to demonstrate phosphorylation by Pka1 of purified Sge1 and Sge1R66S *in vitro* remained inconclusive (data not shown) and will be subject of further investigation.

The loss of effector (*SIX*) gene expression in the *sge1* mutant supports a role of Sge1 as a transcriptional activator controlling the onset of parasitic growth. In *Fol*, these effector genes encode small cysteine rich proteins secreted during colonization of xylem vessels, designated Secreted in xylem (Six) proteins [21],[34]. *SIX1* is only expressed during *in planta* growth and encodes Avr3, as it is required for *I-3* mediated resistance [22],[34]. Six3 is Avr2 and is required for *I-2* mediated resistance [25]. Both Six1/Avr3 and Six3/Avr2 are required for full virulence [23],[25]. No function has yet been assigned to Six2 and Six5. Here, we show that, like Six1, Six2 is highly expressed during *in planta*-mimicking growth conditions (co-cultivation with tomato cells), but *SIX3* and *SIX5*, divergently transcribed from the same promoter region, are expressed in synthetic medium. It could be that Sge1 activity under axenic growth conditions is too low for induction of *SIX1* and *SIX2*, but high enough to induce *SIX3* and *SIX5* expression. The increased *SGE1* expression levels during *in planta* growth may then lead to expression of all *SIX* genes. Currently, a *SGE1* over-expression mutant is being generated in order to determine whether increased *SGE1* expression alone can lead to expression of all *SIX* genes under axenic growth conditions. At the moment, it is not known whether Sge1 influences the expression of the *SIX* genes directly or indirectly, for instance through involvement of other (transcription) factors, nor how *SGE1* expression itself is regulated. Preliminary promoter analysis of the *SIX* genes revealed a potential common motif (unpublished data). The role of this motif in *SIX* gene expression and the question whether Sge1 is able to bind DNA and the *SIX* gene promoter regions in particular remain to be elucidated.

All the genes tested in the *SGE1* deletion mutant (*SIX1*, *SIX2*, *SIX3*, and *SIX5*) were found to be dependent on Sge1 for their expression, even during growth in synthetic medium. Although deletion of individual *SIX* genes (*SIX1* or *SIX3*) only leads to a minor reduction in pathogenicity, the loss of expression of all *SIX* genes in the *sge1* mutant may be the primary cause of the complete non-pathogenic phenotype, due to the inability to suppress host defence responses. Still, loss of pathogenicity upon deletion of *SGE1* may not be due to loss of production of effector proteins only. The majority of pathogenicity genes found in *F. oxysporum* have pleiotropic functions [35],[36]. It is therefore unlikely that expression of these genes is altered in the *sge1* mutant, since this mutant did not display growth defects other than a minor effect in conidiogenesis. One described *F. oxysporum* mutant that is only disturbed in pathogenicity is the *fow2* mutant [37]. Expression of *FOW2* nor that of two other pathogenicity genes, the protein kinase gene *SNF1* [38] and velvet homolog gene *FOXG_00016* [39], is altered in the *SGE1* deletion mutant (unpublished data). However, preliminary results suggest that the expression of the *FTF1* transcription factor gene, implicated to be involved in pathogenicity [40], seems to be altered in the *sge1* mutant (unpublished data). In addition, secondary metabolite profiling revealed that the *sge1* mutant is reduced in the production of fusaric acid, beauvericin and a number of unknown metabolites compared to the wild type (U. Thrane and C.B. Michielse, unpublished data). High concentrations of fusaric acid may contribute to pathogenicity by reducing host resistance [41],[42]. Thus, processes implicated in pathogenesis other than *SIX* gene expression are also altered in the *sge1* mutant, supporting the hypothesis that Sge1 plays a central role during parasitic growth. Deletion of the Sge1 homolog in *Botrytis cinerea*, *BC1G_11680*, also leads to a severely reduced pathogenicity phenotype (C.B. Michielse and P. Tudzynski, unpublished data), indicating that the role of Sge1-like proteins in (plant) pathogenic fungi might be conserved.

The regulation of expression of phase specific genes (*SIX1*, *SIX2*), and genes involved in virulence (*SIX1*, *SIX3*) by Sge1 resembles the functions of Wor1 in *C. albicans* and Ryp1 in *H. capsulatum*. Wor1 has been shown to bind directly to the upstream region of target genes [20]. Interestingly, these target genes all have a large upstream region, indicating that large upstream regions might be a prerequisite for Wor1 binding. Whether this is a common feature shared between Wor1 and Sge1 is still unknown. Future efforts include the identification of additional genes regulated by Sge1 and, eventually, to unravel the network of transcriptional regulators involved in activating the pathogenicity program in *F. oxysporum*. This will also reveal whether Sge1 indeed plays a master role in this network, like its homologs in dimorphic fungi. Given the striking conservation of Sge1 across fungi, we expect that many features of this network will turn out to be similar in pathogens of both plants and animals. Obviously, since Sge1 homologs are also present in non-pathogenic fungi, this regulatory network must serve a fundamental morphological or physiological switch function and pathogens would have adopted this network for adapting to the host environment. 

## Materials and Methods

### Strains, plant material and culture conditions

*Fusarium oxysporum* f. sp. *lycopersici* strain 4287 (race 2; FGSC9935) was used as the parent strain for fungal transformation. It was stored as a monoconidial culture at −80°C and revitalized on potato dextrose agar (PDA, Difco) at 25°C. *Agrobacterium tumefaciens* EHA105 [43] was used for *Agrobacterium*-mediated transformation of *F. oxysporum* and was grown in 2YT medium [44] containing 20 µg/ml rifampicin at 28°C. Introduction of the plasmids into the *Agrobacterium* strain was performed as described by Mattanovich *et al* [45]. *Escherichia coli* DH5 alpha (Invitrogen) was used for construction, propagation, and amplification of the plasmids and was grown in LB medium at 37°C containing either 100 µg/ml ampicillin or 50 µg/ml kanamycin depending on the resistance marker of the plasmid used. Plant line Moneymaker ss590 (Gebr. Eveleens b.v., The Netherlands) was used to assess pathogenicity of *F. oxysporum* strains and transformants. Biocontrol assays were performed on flax (*Linum usitatissimum*) cv. viking using *F. oxysporum* f. sp. *lini* (Foln3, MYA-1201) isolated from a diseased flax plant in French Britany as pathogenic strain.

### Construction of gene replacement and complementation constructs

To generate the *SGE1* disruption construct, p*SGE1*KO, PCR was performed using a BAC clone containing the *SGE1* gene as a template with primer combination FP878–FP879 and FP880–FP881 in order to amplify a 901 bp *Hind*III fragment corresponding to the upstream region and a 1032 bp *Knp*I fragment corresponding to the downstream region, respectively (Table S1). The fragments were sequentially cloned into pPK2*hphgfp* [9] and proper orientation of the inserts was checked by PCR.

The *SGE1* complementation construct, p*SGE1*com, was generated by cloning a 2234 bp *Hind*III fragment containing the *SGE1* ORF including 901 bp upstream and 341 bp downstream sequences into pUC19, resulting in pUC19*SGE1*. The *Hind*III *SGE1* fragment was subsequently transferred from pUC19*SGE1* into pRW1p [24], resulting in p*SGE1*com. The *SGE1* complementation construct carrying a point mutation in the ORF of *SGE1*, p*SGE1*comPM, was generated by replacing a 465 bp *Sac*II/*Bgl*II *SGE1* fragment in p*SGE1*com by a 465 bp *Sac*II/*Bgl*II fragment isolated from the yeast-two-hybrid bait vector pASSGE1PM. This vector was re-isolated from *Saccharomyces cerevisiae* after previously being transformed with pASSGE1. The latter vector was generated by cloning an *Nco*I-*Eco*RI 1011 bp PCR product obtained with the primers FP1484 and FP1485 corresponding to the *SGE1* ORF in pAS2.1 (Clontech).

To generate the *FoPAC2* gene disruption construct, a 1037 bp upstream fragment and a 749 bp downstream fragment was amplified from genomic DNA by PCR with the primer pairs FP1796–FP1797 and FP1798–FP1799, respectively. The PCR products were cloned into pGEM-T Easy (Promega) and, subsequently, the *Kpn*I/*Pac*I upstream and the *Asc*I/*Hind*III downstream fragment were sequentially cloned in pRW2h [24].

The Sge1-fluorescent protein fusion constructs p*SGE1::CFP* and p*SGE1::RFP* were generated in a multi-step approach. First, pUC19*SGE1* was amplified by PCR with the primers FP1120 and FP1121 flanked at the 5′ end by an *Apa*I and a *Spe*I restriction site, respectively. The amplified product was digested with *Apa*I and religated, resulting in pUC19*SGE1*as, containing the *SGE1* ORF with the *Apa*I and *Spe*I preceding the *SGE1* stop codon. Secondly, a CFP [46] and a mRFP [47] fragment were generated by PCR using primer pair FP1122–FP1123 and FP1120–FP1121, respectively. The CFP and RFP PCR products were digested with *Apa*I and *Spe*I and directionally cloned in pUC19*SGE1*as, resulting in pUC*SGE1::CFP* and pUC*SGE1::RFP*, respectively. Finally, a *Hind*III fragment corresponding to each fusion cassette was cloned into *Hind*III digested pRW1p and proper orientation of the fragments was checked by PCR. The gpdAH2B::GFP expression cassette was isolated as a *Xba*I/*Nhe*I fragment from pH2B::GFP (kind gift from Dr. Ram, Leiden, The Netherlands) and cloned into *Xba*I/*Nhe*I digested pRW2h [24] to generate pRWH2B::GFP.

### Fungal transformation and disease assay

*Agrobacterium*-mediated transformation of *F. oxysporum* f. sp. *lycopersici* was performed as described by Mullins and Chen [48] with minor adjustments [49]. Depending on the selection marker used, transformants were selected on Czapek Dox agar (CDA, Oxoid) containing 100 µg/ml Hygromycin (Duchefa) or on CDA containing 0.1 M TrisHCl pH 8 and 100 µg/ml Zeocin (InvivoGen).

Plant infection was performed using 9 to 11 days old seedlings (Moneymaker ss590), following the root-dip inoculation method [50]. Disease index was scored and statistical analysis performed as described earlier [9].

### Analysis of conidiogenesis, conidia germination, carbon source utilization and cellophane penetration

For quantization of microconidia production, five independent experiments were performed, each with five replicates. Microconidia were harvested after five days of growth in 50 ml minimal medium (3% sucrose, 10 mM KNO_3_ and 0.17% yeast nitrogen base without amino acids and ammonia) and 10^6^ spores were used to inoculate 100 ml minimal medium. After five days the microconidia produced were harvested and counted in a Bürker-Türk haemocytometer. The isolated conidia were also used to determine germination rates. To this end 600 spores were added into a 6-wells plate with each well containing 250 µl PDA and incubated overnight at 4°C, then transferred to 25°C and germinated conidia were counted after six hours of incubation. Macroconidia development was analyzed in liquid carboxymethyl cellulose medium as described by Ohara and Tsuge [30]. Conidia were fixed in 0.4% *p*-formaldehyde and stained with Hoechst 33342 (250 µg/ml) and calcofluor white (25 µg/ml) to visualize nuclei and cell walls, respectively. Stained cells were observed with a BX50 fluorescence microscope and a U-MWU filter (Olympus).

Carbon-source utilization was tested in a BIOLOG FF MicroPlate (BIOLOG). A conidial suspension (10^4^ conidia in 150 µl) of each strain was inoculated in each well of the plate and incubated at 25°C. The absorbance of each well at 600 nm was measured with a microtiter plate reader (Packerd Spectra Count) after 1, 2, 3 and 4 days of incubation.

The cellophane penetration assay was performed as described [18], with minor adjustments. CDA was used as basal medium in the assay and was inoculated with 10^5^ conidia. After incubation of 5 days at 25°C, the cellophane was removed and after a subsequent incubation of 2 days at 25°C fungal growth was scored.

### Southern analysis

Genomic DNA was isolated as described by Kolar *et al* [51] with minor adjustments [9]. For Southern analysis, 10 µg genomic DNA of each transformant was digested with *Acc*65I or *Bgl*II for *SGE1* or *FoPAC2* transformants, respectively, and incubated overnight at 37°C. The samples were loaded on a 1% 0.5× Tris-borate/EDTA gel and run for 18 hours at 45 V. The digested DNA was transferred to Hybond-N+ (Amersham Pharmacia) as described by Sambrook *et al* [44]. The probes used for Southern analysis are: (1) a 432 bp *SGE1* upstream fragment obtained by PCR with primers FP842 and FP1174 (Table S1) and (2) a 488 bp *FoPAC2* downstream fragment obtained by PCR with primers FP2198 and FP 2199. The DecaLabel™ DNA Labelling Kit (Fermentas) was used to label probes with [α-^32^P]dATP. Hybridization was done overnight at 65°C in 0.5 M sodium phosphate buffer, pH 7.2, containing 7% SDS and 1 mM EDTA. Blots were washed with 0.2× SSC, 0.1% SDS. Hybridization signals were visualized by phosphorimaging (Molecular Dynamics).

### RT and quantitative PCR

Samples for expression analysis were obtained by adding 0,5 ml of 10^7^ conidia/ml of wild type, *SGE1* deletion or *SGE1* complementation strain to 4,5 ml of a one week old MSK8 cell culture grown in BY-medium [52]. After 24 hours of incubation at 22°C, the material was harvested, washed two times with sterile water and freeze-dried. Total RNA was isolated using Trizol (Invitrogen) and prior to cDNA synthesis the RNA was treated with DnaseI (Fermentas). First strand cDNA was synthesized with M-MuLV reverse transcriptase following the instruction of the manufacturer (Fermentas). Supertaq (SphaeroQ), 1 µl of the cDNA reaction and primers FP1999/FP2000, FP998/FP1001, FP962/FP963, FP1993/FP1994, FP2131/FP2132 and FP157/FP158 (Table S1) to detect *SIX1* (CAE55879), *SIX2* (CAE55868), *SIX3* (CAJ83999), *SIX5* (ACN87967), *SGE1* (*FOXG_10510*) and *FEM1* (AAL47843) transcripts, respectively, were used in the RT-PCR.

Quantitative PCR (qPCR) was performed with Platinum SYBR Green qPCR (Invitrogen) using a 7500 Realtime PCR System (Applied Biosystems). To quantify mRNA levels of *SGE1* and of the constitutively expressed *EF-1α* gene, we used primers FP2131/FP2132 and FP2029/FP2030, respectively (Table S1). *EF-1α* was used to calculate the relative expression levels of *SGE1* in axenic and MSK8 cultures infected with *F. oxysporum* wild type, *SGE1* deletion or *SGE1* complementation mutants. Real time PCR primer efficiencies were calculated using LinRegPCR [53] and relative expression levels were calculated according to the comparative C(t) method [54].

### Microscopic analysis

Sge1::mRFP, Sge1::CFP and H2B::GFP fusion proteins were visualized using a BX50 fluorescence microscope with the appropriate excitation and emission filters for CFP, mRFP and GFP (Olympus). For this purpose, 10 µl of five days old minimal medium cultures was spotted onto glass slides.

A Nikon A1 microscope was used to monitor tomato root infection by the wild type and *SGE1* deletion mutant. Excitation was provided for the GFP signal with an Argon (488 nm) laser (emission: 405–455 nm) and for the root auto-fluorescence with an UV diode (405 nm) laser (emission: 420–470 nm). Images were line-sequential scanned (2 µm slices, 1024×1024 pixels) using water objective plan fluor 20× Imm, 0.75 NA. Pictures were analyzed with the Nikon NIS and ImageJ software. For preparation of the slides, 9 days old tomato seedlings were carefully removed from the potting soil, rinsed with water and intact roots were inoculated with 20 ml tap water containing 10^7^ conidia/ml and incubated for 4 days at room temperature in a petridish. The roots were rinsed with water, cut from the hypocotyl, placed in a drop of water on a glass slide and covered with a cover glass. A bridge mounted on the glass slide prevented squashing of the root material.

### Inoculum preparation and biocontrol assay

Inocula were prepared as described [55], except that minimal liquid medium [56] in which sucrose was replaced by glucose (5 g/l) and sodium nitrate by ammonium tartrate (1 g/l) was used instead of Malt medium. A heat treated (100°C for 1 h) silty-loam soil from Dijon (35.1% clay, 47% loam, 15.1% sand, and 1.22% organic C [pH = 6.9]) was added to module trays containing 96 wells each of 50 ml. To prevent contamination between treatments, only every second row of wells was filled with soil. The soil in each well was inoculated with 5 mL of conidia suspension. The concentration of the conidia suspensions were adjusted to obtain the following inoculum densities: 1×10^4^ conidia ml^−1^ of soil for the pathogenic strain Foln3 and 1×10^6^ conidia ml^−1^ of soil for the two others strains. The soil surface was covered with a thin layer of calcinated clay granules (Oil Dri Chem-Sorb, Brenntag Bourgogne, Montchanin, France) and three seeds of flax, cv. viking, were sown in each pot. A thin layer of Chem-Sorb was used to cover the seeds. Plants were grown in a growth chamber in the first 2 weeks; the growing conditions were 8 h 15°C N/16 h 17°C D, with a light intensity of 33 µE m^−2^ s^−1^. The plants were thinned to one plant per pot, and from week 3 the temperature was kept at 22°C N/25°C D. There were three replicates of 16 individual plants per treatment, randomly arranged, and the experiment was replicated. Plants were watered every day, and once a week water was replaced by a 500-fold dilution of a commercial nutrient stock solution (“Hydrodrokani AO”, Hydro Agri, Nanterre, France). Plants showing characteristic symptoms of yellowing were recorded twice a week and removed. To compare the ability of strains to induce disease or, on the contrary, to protect the plant against wilt, ANOVA was performed on Area under the Disease Progress Curves followed by Newman and Keuls test at the probability of 95%.

## Supporting Information

Table S1 Primers used in this study.
(0.01 MB PDF)

Figure S1 Analysis of transformants deleted for *SGE1*. A knock-out construct containing a hygromycin-GFP expression cassette flanked by 901 bp up- and 1032 bp downstream sequences of SGE1 was introduced in the wild type strain by *Agrobacterium*-mediated transformation. A) Schematic representation of the knock-out strategy for *SGE1* drawn to scale. The arrow heads indicate the positions of the original T-DNA insertions. The small arrows represent the primers used to check homologous recombination (a and b) and the absence of the open reading frame (c and d). B) Verification of homologous recombination by PCR using primers a and b. C) Verification of the absence of the *SGE1* open reading frame by PCR using primers c and d. The 1 kb DNA ladder of Fermentas (www.fermentas.com) is used as a marker. −, negative control. +, positive control (genomic DNA). The figures are composed from different parts of an ethidium bromide gel, which results in minor colour differences.
(0.41 MB PDF)

Figure S2 Analysis of transformants complemented with *SGE1*. A complementation construct containing a phleomycin expression cassette and the *SGE1* gene including 901 bp up- and 341 bp downstream sequences was introduced in the *SGE1* knock-out mutant #32 by *Agrobacterium*-mediated transformation. A) Schematic representation of the complementation strategy for *SGE1* drawn to scale. The small arrows represent the primers used to check the presence of the open reading frame (c and d). B) Verification of the presence of the *SGE1* ORF by PCR using primers c and d. The 1 kb DNA ladder of Fermentas (www.fermentas.com) is used as a marker. −, negative control.
(0.09 MB PDF)

Figure S3 Southern analysis of the *SGE1* deletion and complementation mutants. Southern analysis was performed to verify correct homologous recombination at the *SGE1* locus in the *SGE1* disruptants and complemented strains. To this end, chromosomal DNA of the various mutants was digested with *Acc65*I, blotted and hybridized with a 432 bp probe corresponding to the *SGE1* promoter. The *SGE1* locus of the wild type strain is visible as a 1.6 kb fragment corresponding to the *SGE1* upstream region and to the 5′ part of the *SGE1* ORF. In the 5G2 insertional mutagenesis mutant, the *SGE1* ORF is disrupted due to a T-DNA insertion (see Figure S1A). As a result the 1.6 kb fragment observed in a wild type situation is replaced by a 1.8 kb fragment corresponding to a part of the *SGE1* promoter region and the hygromycin expression cassette present on the T-DNA. In the *SGE1* disruption mutants introduction of the gene replacement cassette by homologous recombination led to the expected replacement of the 1.6 kb fragment with a fragment containing part of the *SGE1* upstream region and the gene disruption cassette which should be larger than 4.9 kb. Introduction of the *SGE1* complementation cassette, including the SGE1::FP fusion protein complementation cassettes, by homologous recombination restored the wild type *SGE1* locus. WT, wild type. 5G2, insertional mutagenesis mutant 5G2. KO, *SGE1* knock-out mutants. com, *SGE1* complementation mutants. CC, *SGE1::CFP* complementation mutants. RC, *SGE1::RFP* complementation mutants.
(0.05 MB PDF)

Figure S4 Analysis of transformants deleted for *FoPAC2*. A knock-out construct containing a hygromycin expression cassette flanked by 1037 bp up- and 749 bp downstream sequences of *FoPAC2* was introduced in the wild type strain by *Agrobacterium*-mediated transformation. A) Schematic representation of the knock-out strategy for *FoPAC2* drawn to scale. The arrow head indicates the position of the original T-DNA insertion. The small arrows represents the primers used to check homologous recombination (a and b) and the absence of the open reading frame (c and d). B) Verification of the absence of the *FoPAC2* open reading frame by PCR using primers c and d. C) Verification of homologous recombination by PCR using primers a and b. D) Southern analysis of the *FoPAC2* deletion mutants. Chromosomal DNA was digested with *Bgl*II, blotted and hybridized with a 488 bp probe corresponding to upstream region. The *FoPAC2* locus of the wild type strain is visible as a 7.0 kb fragment. In the 30C11 insertional mutagenesis mutant, the *FoPAC2* ORF is disrupted due to a T-DNA insertion (see Figure S4A). As a result the 7.0 kb fragment observed in a wild type situation is replaced by a 12.3 kb fragment corresponding to a part of the *FoPAC2* upstream region and the hygromycin expression cassette present on the T-DNA. In the *FoPAC2* disruption mutants introduction of the gene replacement cassette by homologous recombination led to the expected replacement of the 7.0 kb fragment with a 11.4 kb fragment containing part of the *FoPAC2* upstream region and the gene disruption cassette. The 1 kb DNA ladder of Fermentas (www.fermentas.com) is used as a marker. −, negative control. +, positive control (genomic DNA). Panels B and C are composed of different parts of an ethidium bromide gel, which results in minor colour differences.
(0.15 MB PDF)

Figure S5 The *SGE1* deletion mutant is not impaired in carbon source utilization. To analyze carbon utilization of the *sge1* mutant, BIOLOG FF MicroPlates containing in each well a different carbon source were used. A conidial suspension (10^4^ conidia in 150 µl) of the wild type or the *SGE1* deletion stain was inoculated in each well and incubated at 25°C. The absorbance of each well at 600 nm was measured with a microtiter plate reader (Packerd Spectra Count) after 4 days of incubation. The ratios were calculated by dividing the measured values of the mutant strain by those of the wild type strain. Only values higher than 1.5 or lower than 0.5 were marked (boxed) as carbon sources on which the *sge1* mutant appeared to display a different growth rate than the wild type strain in this experiment. An additional plate assay containing these carbon sources showed that these differences were not reproducible (data not shown).
(0.40 MB TIF)

Figure S6 Micro- and macroconidia of the *SGE1* deletion mutant are morphologically indistinguishable from wild type. Micro- and macroconidia development was analyzed in liquid carboxymethyl cellulose medium. Conidia were fixed in 0.4% *p*-formaldehyde and stained with Hoechst 33342 (250 µg/ml) and calcofluor white (25 µg/ml) to visualize nuclei and cell walls, respectively. The left panel depicts a phase contrast recording and the right panel depicts a UV-exposed recording of conidia of the *sge1* mutant.
(1.38 MB PDF)

Figure S7 *SGE1* is not essential for superficial root colonization. The root colonization behaviour of the *SGE1* deletion mutant was determined by binocular microscopy. Nine to eleven days old tomato seedlings were inoculated with wild type or an *SGE1* deletion mutant spore suspension and root colonization was determined after two to five days after inoculation. Patches of colonization were already visible after three days. The left panel depicts phase contrast recordings of a GFP-expressing virulent strain (38H3) and of the *SGE1* deletion mutant. The right panel depicts UV-exposed recordings.
(0.82 MB PDF)

Figure S8 The *SGE1* mutant is not impaired in cellophane penetration. The capacity of the *SGE1* deletion strain to penetrate cellophane was determined using a cellophane penetration assay. CDA medium covered by a cellophane sheet was inoculated with a drop containing 10^5^ conidia. After incubation of 5 days at 25°C (left panel), the cellophane was removed and after a subsequent incubation of 2 days at 25°C fungal growth of both the wild type and the *SGE1* deletion mutant was clearly observed (right panel).
(0.25 MB PDF)

Figure S9 The *sge1* mutant is impaired in extensive *in planta* growth. To determine whether the *SGE1* deletion mutant is able to growth within xylem vessels, tomato seedlings were inoculated with the wild type, *SGE1* knock-out or the *SGE1* complementation strain and potted into soil according to the bioassay procedure. One week after inoculation the hypocotyl was cut in slices of several millimeters which were placed on rich (PDA) medium. *F. oxysporum* outgrowth was observed from the hypocotyl pieces previously inoculated with the wild type and the *SGE1* complementation strain, but not from the hypocotyl pieces previously inoculated with the *SGE1* knock-out mutant.
(0.26 MB PDF)

Figure S10 Pathogenicity is partially restored in *SGE1::FP* complementation strains. Nine to eleven days old tomato seedlings were inoculated with fungal spore suspensions following the root-dip inoculation method and the disease index (ranging from 0 healthy plant to 4 severely diseased plant/dead plant) was scored after three weeks. Average disease index of 20 plants three weeks after mock inoculation (H_2_O) or inoculation with a *SGE1* deletion mutant (*SGE1*KO32), *SGE1* (*SGE1*com28), *SGE1::CFP* (*SGE1*comCC4, 5, and 10) and *SGE1::RFP* (*SGE*comRC2, 6, and 11) complementation mutants or wild type (4287). Error bars indicate standard deviation and capitals define statistically different groups (ANOVA, p = 0.95).
(0.03 MB PDF)

Figure S11 Analysis of transformants complemented with *SGE1PM*. A complementation construct containing a phleomycin expression cassette and the *SGE1PM* gene, encoding Sge1R66S, including 901 bp up- and 341 bp downstream sequences, was introduced in the *SGE1* knock-out mutant #32 by *Agrobacterium*-mediated transformation. A) Verification of the presence of the *SGE1* ORF by PCR using primers c and d (see Figure S1A). B) Verification of homologous recombination by PCR using primers a and b (see Figure S2). The 1 kb DNA ladder of Fermentas (www.fermentas.com) is used as a marker. −, negative control. Deletion mutant *SGE1*KO32 and complementation mutant *SGE1*com28 were used as a negative and positive control, respectively.
(0.34 MB PDF)
