# Supplementary material for: The Nuclear Protein Sge1 of Fusarium oxysporum Is Required for Parasitic Growth
Source: PLoS Pathog. 2009 Oct 23;5(10):e1000637. doi: 10.1371/journal.ppat.1000637 (PMC2762075; doi:10.1371/journal.ppat.1000637)
Supplement: Table S1 — Primers used in this study. (0.01 MB PDF) [file ppat.1000637.s001.pdf]

Table S1. Primers used in this study.

primers	sequence (5’-3’)
FP157	atgaagtacactctcgctacc
FP158	ggtgaaagtgaaagagtcacc
FP842	gagttcgagagctcaactttc
FP878	ttactaggtacctacctgctc
FP879	aaaaagcttttggaagggatagccttcaag
FP880	agctcctgacaggtcagaac
FP881	aaaggtaccgggaatgaacgttttctgcag
FP962	tgagcgggctggcaattc
FP963	caatcctctgagatagtaag
FP998	atgctcttcaaaatcgcgtg
FP1001	caacgccgtttgaataagca
FP1120	aaagggcccccaccacgtctgaccataag
FP1121	aaagggcccaaaactagttagaacgtgcttatatagagcttc
FP1122	aaagggcccatggtgagcaagggcgagga
FP1123	aaaactagtcttgtacagctcgtccatgc
FP1174	gtggcatggttccagacatcttg
FP1484	catgccatgggaatgtctggaaccatgccactc
FP1485	ccggaattcctaccaccacgtctgaccata
FP1796	aaggcgcgcctcacctgcgccaactcg
FP1797	gctctagaaggagtctgtagttgctcg
FP1798	ggttaattaacaaatgcgactacaaagaatctc
FP1799	ggggtacccctaatccatcgtgtctctg
FP1993	gcgcttcgagtacatctctg
FP1994	ctaggccgcatcacaataga
FP1999	ctcaagaggctgcggttc
FP2000	caagttgcgcgatatgtgtt
FP2029	cggtaagggttccttcaagt
FP2030	tgaccgggagcgtcgatga
FP2131	tgaaggctatcccttccaa
FP2132	ccagagagacatgcctcaaa
FP2198	caatgcactgcgtacgtac
FP2199	caatctcacgagaccaattc

Restriction sites are underlined.
