# Supplementary figures and images for: The Nuclear Protein Sge1 of Fusarium oxysporum Is Required for Parasitic Growth
Source: PLoS Pathog. 2009 Oct 23;5(10):e1000637. doi: 10.1371/journal.ppat.1000637 (PMC2762075; doi:10.1371/journal.ppat.1000637)

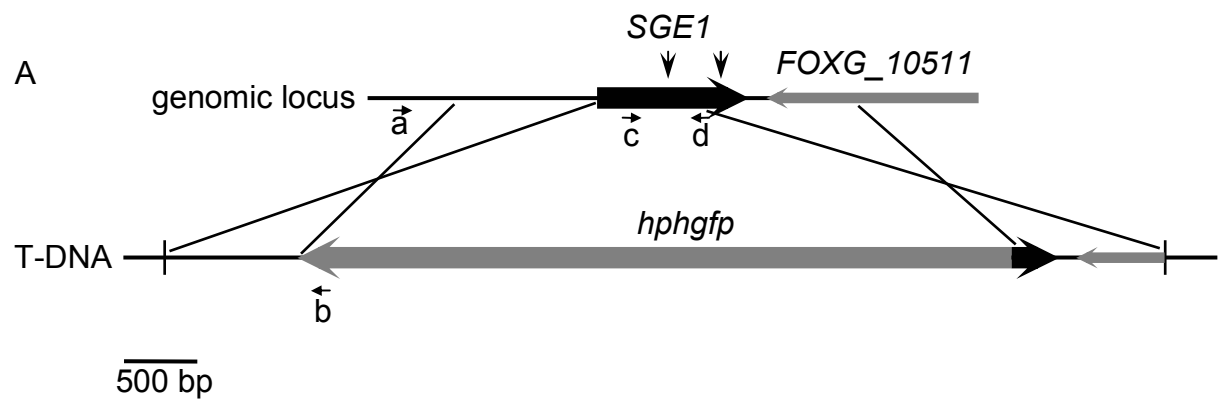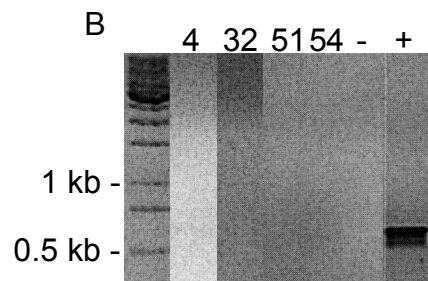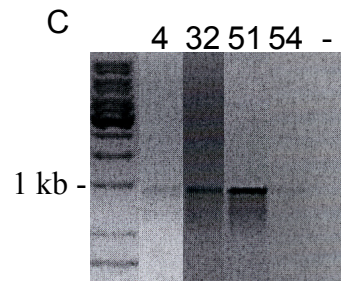

Supplement: Figure S1 — Analysis of transformants deleted for SGE1. A knock-out construct containing a hygromycin-GFP expression cassette flanked by 901 bp up- and 1032 bp downstream sequences of SGE1 was introduced in the wild type strain by Agrobacterium-mediated transformation. A) Schematic representation of the knock-out strategy for SGE1 drawn to scale. The arrow heads indicate the positions of the original T-DNA insertions. The small arrows represent the primers used to check homologous recombination (a and b) and the absence of the open reading frame (c and d). B) Verification of homologous recombination by PCR using primers a and b. C) Verification of the absence of the SGE1 open reading frame by PCR using primers c and d. The 1 kb DNA ladder of Fermentas (www.fermentas.com) is used as a marker. −, negative control. +, positive control (genomic DNA). The figures are composed from different parts of an ethidium bromide gel, which results in minor colour differences. (0.41 MB PDF) [file ppat.1000637.s002.pdf]

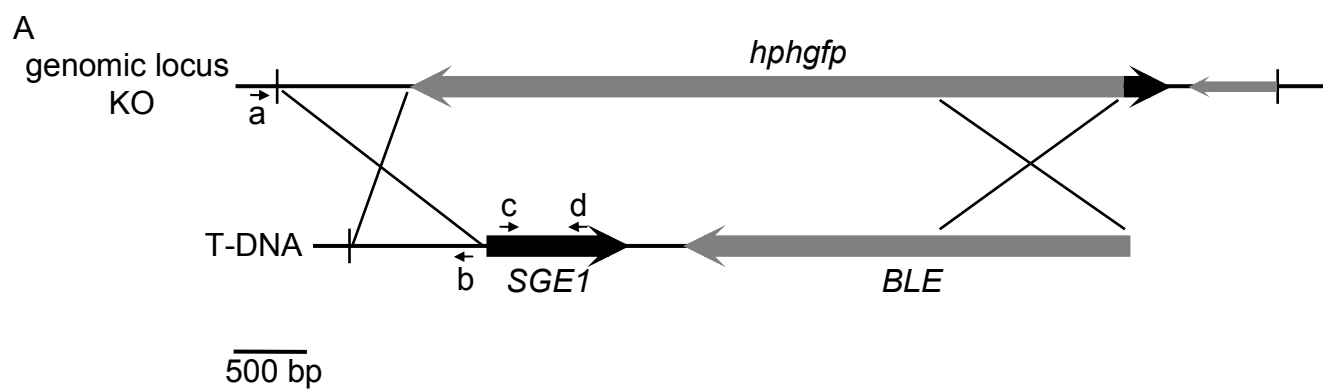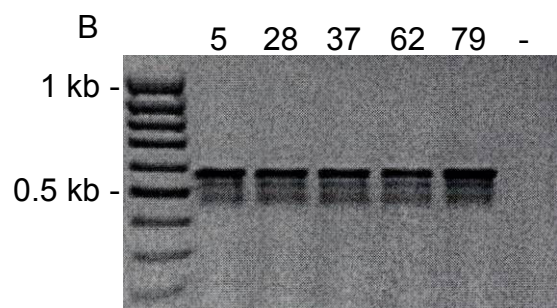

Supplement: Figure S2 — Analysis of transformants complemented with SGE1. A complementation construct containing a phleomycin expression cassette and the SGE1 gene including 901 bp up- and 341 bp downstream sequences was introduced in the SGE1 knock-out mutant #32 by Agrobacterium-mediated transformation. A) Schematic representation of the complementation strategy for SGE1 drawn to scale. The small arrows represent the primers used to check the presence of the open reading frame (c and d). B) Verification of the presence of the SGE1 ORF by PCR using primers c and d. The 1 kb DNA ladder of Fermentas (www.fermentas.com) is used as a marker. −, negative control. (0.09 MB PDF) [file ppat.1000637.s003.pdf]

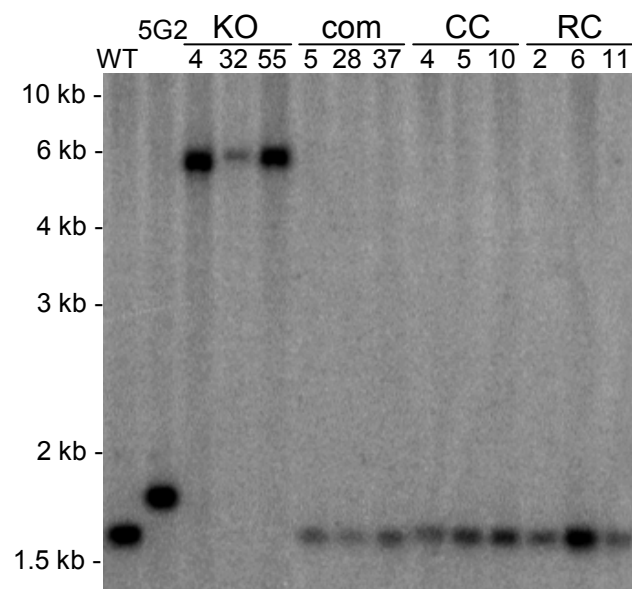

Supplement: Figure S3 — Southern analysis of the SGE1 deletion and complementation mutants. Southern analysis was performed to verify correct homologous recombination at the SGE1 locus in the SGE1 disruptants and complemented strains. To this end, chromosomal DNA of the various mutants was digested with Acc65I, blotted and hybridized with a 432 bp probe corresponding to the SGE1 promoter. The SGE1 locus of the wild type strain is visible as a 1.6 kb fragment corresponding to the SGE1 upstream region and to the 5′ part of the SGE1 ORF. In the 5G2 insertional mutagenesis mutant, the SGE1 ORF is disrupted due to a T-DNA insertion (see Figure S1A). As a result the 1.6 kb fragment observed in a wild type situation is replaced by a 1.8 kb fragment corresponding to a part of the SGE1 promoter region and the hygromycin expression cassette present on the T-DNA. In the SGE1 disruption mutants introduction of the gene replacement cassette by homologous recombination led to the expected replacement of the 1.6 kb fragment with a fragment containing part of the SGE1 upstream region and the gene disruption cassette which should be larger than 4.9 kb. Introduction of the SGE1 complementation cassette, including the SGE1::FP fusion protein complementation cassettes, by homologous recombination restored the wild type SGE1 locus. WT, wild type. 5G2, insertional mutagenesis mutant 5G2. KO, SGE1 knock-out mutants. com, SGE1 complementation mutants. CC, SGE1::CFP complementation mutants. RC, SGE1::RFP complementation mutants. (0.05 MB PDF) [file ppat.1000637.s004.pdf]

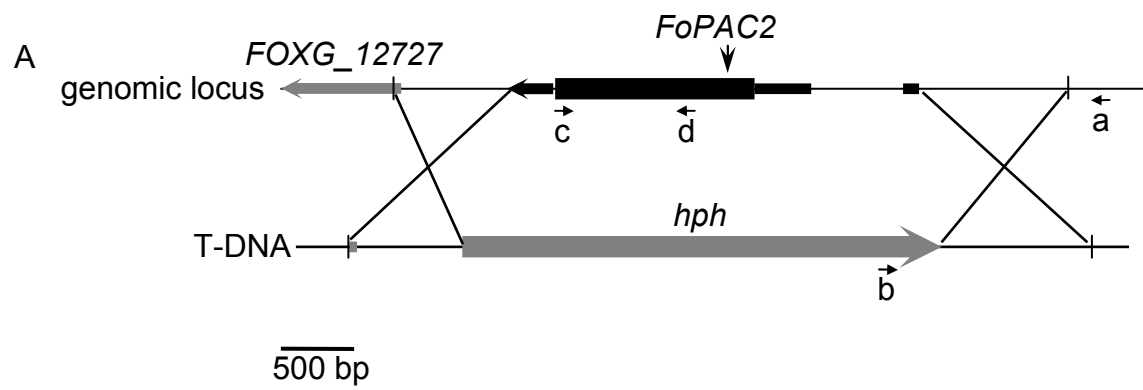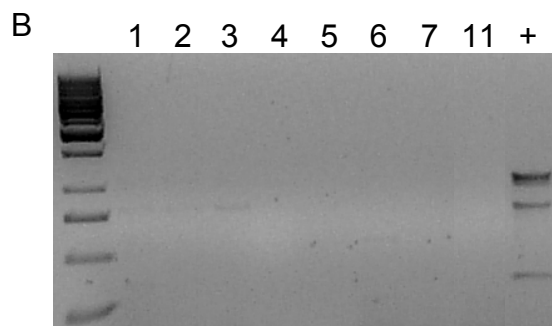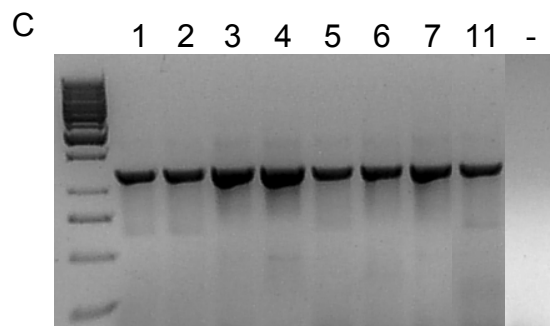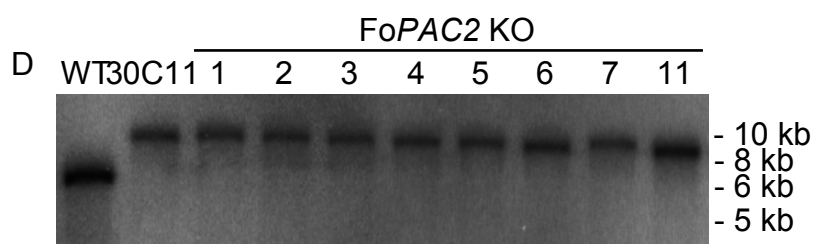

Supplement: Figure S4 — Analysis of transformants deleted for FoPAC2. A knock-out construct containing a hygromycin expression cassette flanked by 1037 bp up- and 749 bp downstream sequences of FoPAC2 was introduced in the wild type strain by Agrobacterium-mediated transformation. A) Schematic representation of the knock-out strategy for FoPAC2 drawn to scale. The arrow head indicates the position of the original T-DNA insertion. The small arrows represents the primers used to check homologous recombination (a and b) and the absence of the open reading frame (c and d). B) Verification of the absence of the FoPAC2 open reading frame by PCR using primers c and d. C) Verification of homologous recombination by PCR using primers a and b. D) Southern analysis of the FoPAC2 deletion mutants. Chromosomal DNA was digested with BglII, blotted and hybridized with a 488 bp probe corresponding to upstream region. The FoPAC2 locus of the wild type strain is visible as a 7.0 kb fragment. In the 30C11 insertional mutagenesis mutant, the FoPAC2 ORF is disrupted due to a T-DNA insertion (see Figure S4A). As a result the 7.0 kb fragment observed in a wild type situation is replaced by a 12.3 kb fragment corresponding to a part of the FoPAC2 upstream region and the hygromycin expression cassette present on the T-DNA. In the FoPAC2 disruption mutants introduction of the gene replacement cassette by homologous recombination led to the expected replacement of the 7.0 kb fragment with a 11.4 kb fragment containing part of the FoPAC2 upstream region and the gene disruption cassette. The 1 kb DNA ladder of Fermentas (www.fermentas.com) is used as a marker. −, negative control. +, positive control (genomic DNA). Panels B and C are composed of different parts of an ethidium bromide gel, which results in minor colour differences. (0.15 MB PDF) [file ppat.1000637.s005.pdf]

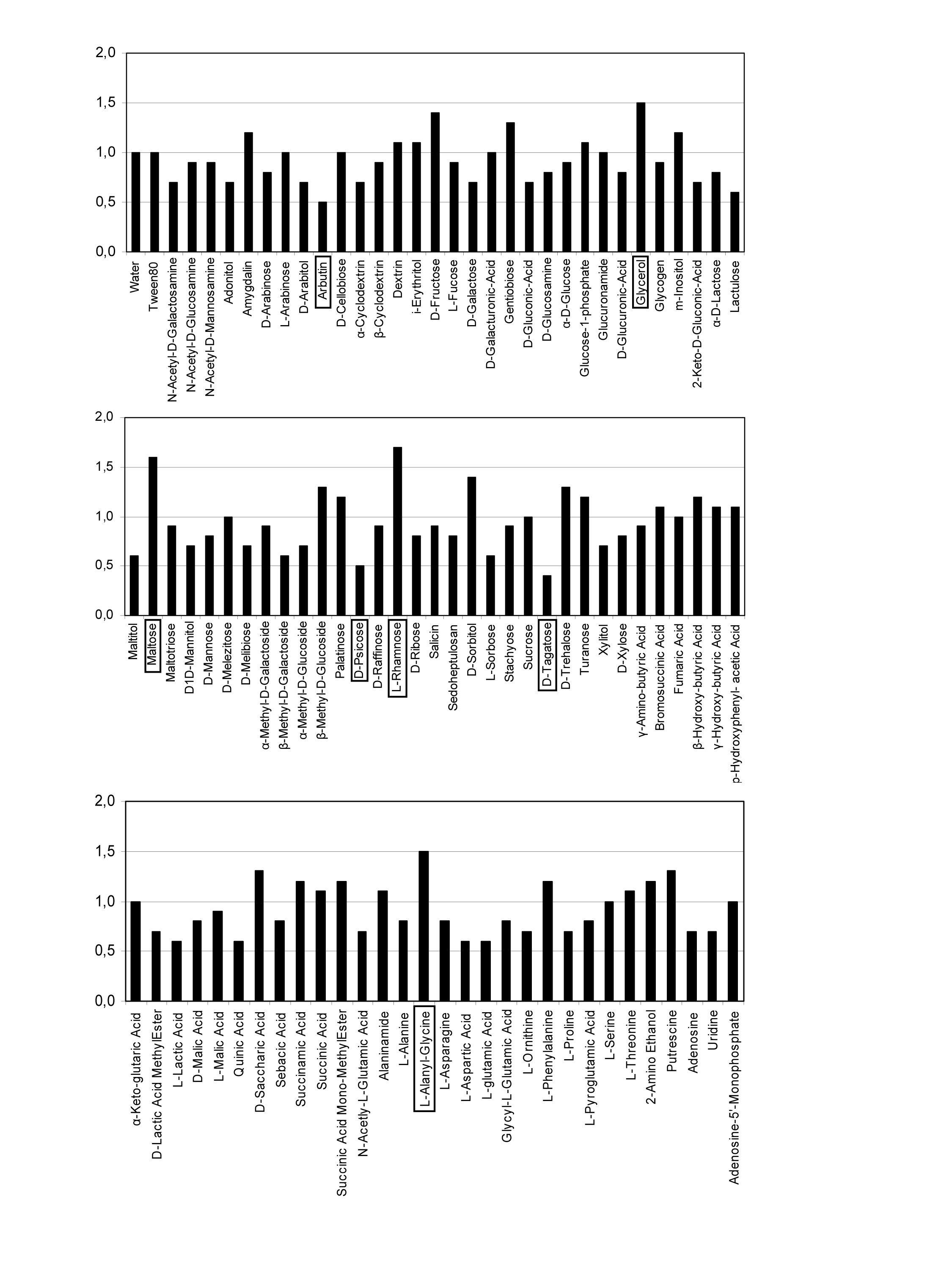

Supplement: Figure S5 — The SGE1 deletion mutant is not impaired in carbon source utilization. To analyze carbon utilization of the sge1 mutant, BIOLOG FF MicroPlates containing in each well a different carbon source were used. A conidial suspension (104 conidia in 150 µl) of the wild type or the SGE1 deletion stain was inoculated in each well and incubated at 25°C. The absorbance of each well at 600 nm was measured with a microtiter plate reader (Packerd Spectra Count) after 4 days of incubation. The ratios were calculated by dividing the measured values of the mutant strain by those of the wild type strain. Only values higher than 1.5 or lower than 0.5 were marked (boxed) as carbon sources on which the sge1 mutant appeared to display a different growth rate than the wild type strain in this experiment. An additional plate assay containing these carbon sources showed that these differences were not reproducible (data not shown). (0.40 MB TIF) [file ppat.1000637.s006.tif]

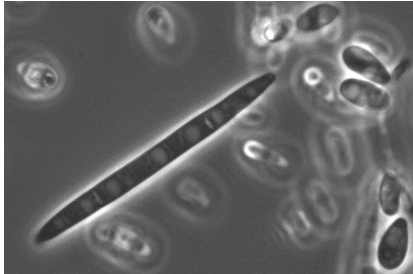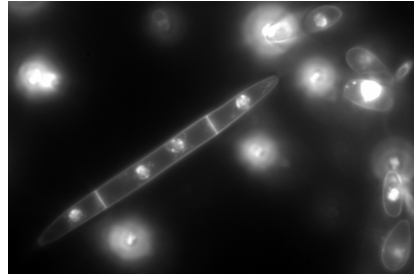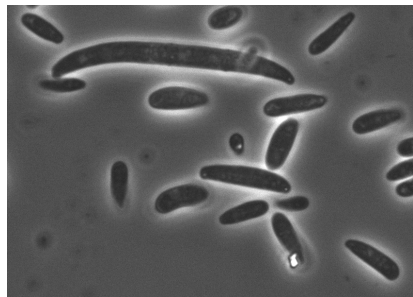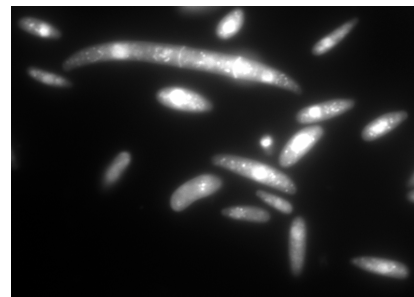

phase contrast

UV exposed

Supplement: Figure S6 — Micro- and macroconidia of the SGE1 deletion mutant are morphologically indistinguishable from wild type. Micro- and macroconidia development was analyzed in liquid carboxymethyl cellulose medium. Conidia were fixed in 0.4% p-formaldehyde and stained with Hoechst 33342 (250 µg/ml) and calcofluor white (25 µg/ml) to visualize nuclei and cell walls, respectively. The left panel depicts a phase contrast recording and the right panel depicts a UV-exposed recording of conidia of the sge1 mutant. (1.38 MB PDF) [file ppat.1000637.s007.pdf]

38H3

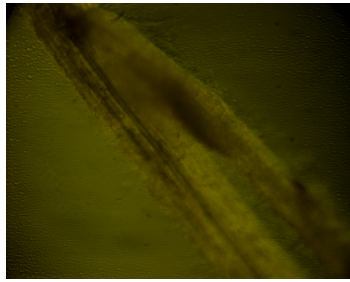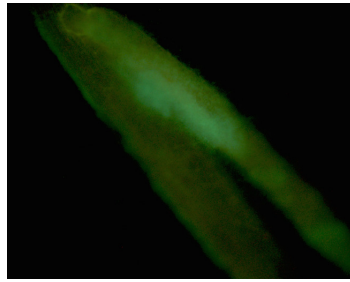

$\Delta SGE1$

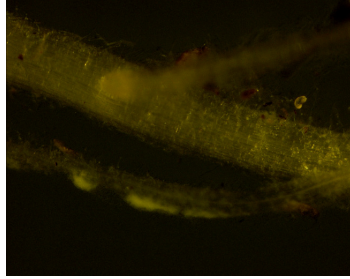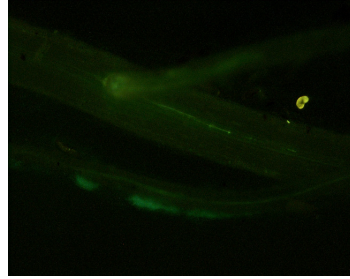

$\Delta SGE1$

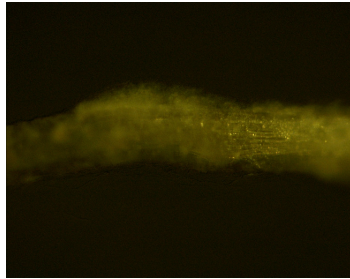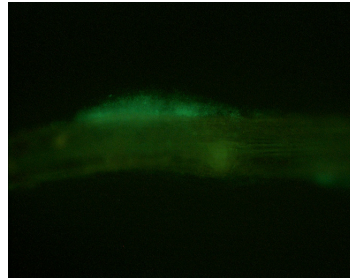

phase contrast

UV exposed

Supplement: Figure S7 — SGE1 is not essential for superficial root colonization. The root colonization behaviour of the SGE1 deletion mutant was determined by binocular microscopy. Nine to eleven days old tomato seedlings were inoculated with wild type or an SGE1 deletion mutant spore suspension and root colonization was determined after two to five days after inoculation. Patches of colonization were already visible after three days. The left panel depicts phase contrast recordings of a GFP-expressing virulent strain (38H3) and of the SGE1 deletion mutant. The right panel depicts UV-exposed recordings. (0.82 MB PDF) [file ppat.1000637.s008.pdf]

before  
cellophane removal

2 days after  
cellophane removal

4287

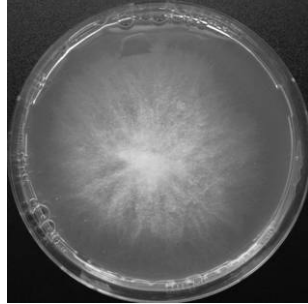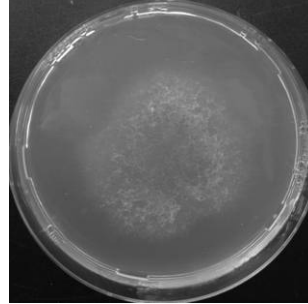

*SGE1* KO32

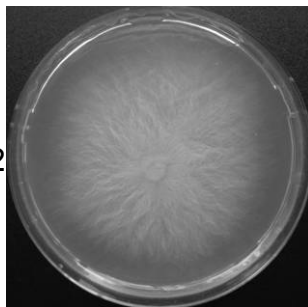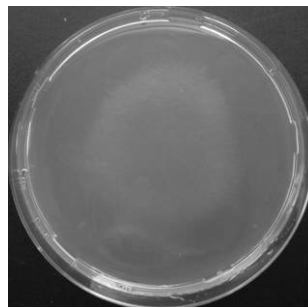

Supplement: Figure S8 — The SGE1 mutant is not impaired in cellophane penetration. The capacity of the SGE1 deletion strain to penetrate cellophane was determined using a cellophane penetration assay. CDA medium covered by a cellophane sheet was inoculated with a drop containing 105 conidia. After incubation of 5 days at 25°C (left panel), the cellophane was removed and after a subsequent incubation of 2 days at 25°C fungal growth of both the wild type and the SGE1 deletion mutant was clearly observed (right panel). (0.25 MB PDF) [file ppat.1000637.s009.pdf]

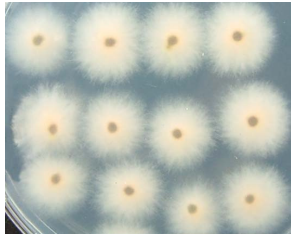

4287

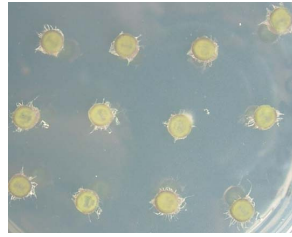

*SGE1* KO

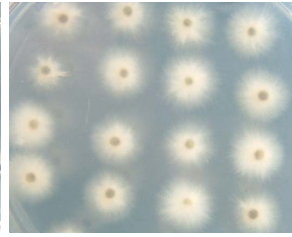

*SGE1* com

Supplement: Figure S9 — The sge1 mutant is impaired in extensive in planta growth. To determine whether the SGE1 deletion mutant is able to growth within xylem vessels, tomato seedlings were inoculated with the wild type, SGE1 knock-out or the SGE1 complementation strain and potted into soil according to the bioassay procedure. One week after inoculation the hypocotyl was cut in slices of several millimeters which were placed on rich (PDA) medium. F. oxysporum outgrowth was observed from the hypocotyl pieces previously inoculated with the wild type and the SGE1 complementation strain, but not from the hypocotyl pieces previously inoculated with the SGE1 knock-out mutant. (0.26 MB PDF) [file ppat.1000637.s010.pdf]

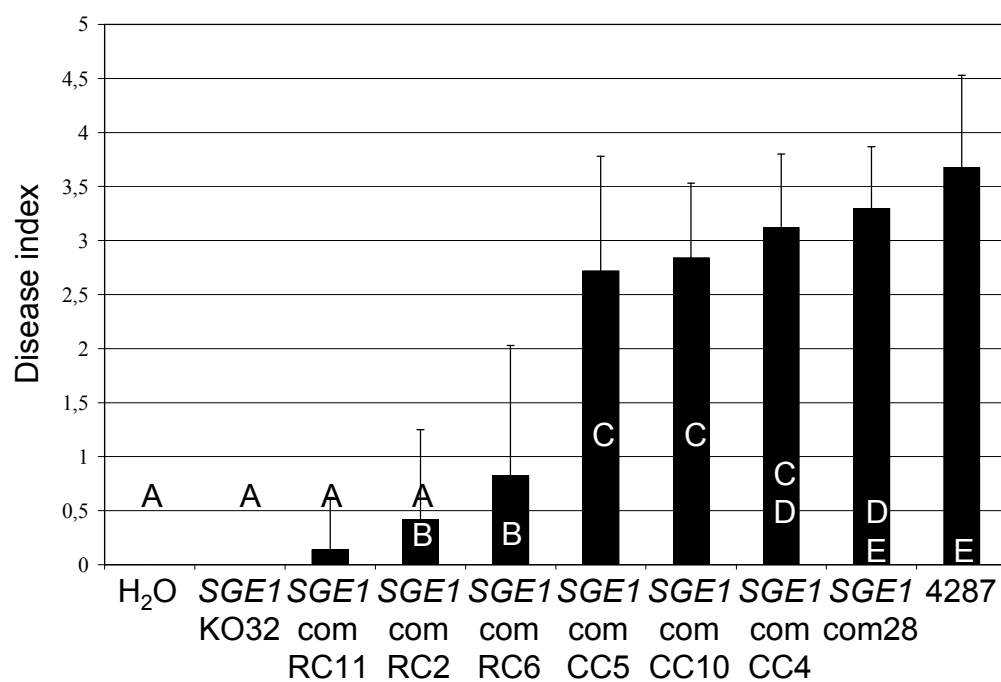

Supplement: Figure S10 — Pathogenicity is partially restored in SGE1::FP complementation strains. Nine to eleven days old tomato seedlings were inoculated with fungal spore suspensions following the root-dip inoculation method and the disease index (ranging from 0 healthy plant to 4 severely diseased plant/dead plant) was scored after three weeks. Average disease index of 20 plants three weeks after mock inoculation (H2O) or inoculation with a SGE1 deletion mutant (SGE1KO32), SGE1 (SGE1com28), SGE1::CFP (SGE1comCC4, 5, and 10) and SGE1::RFP (SGEcomRC2, 6, and 11) complementation mutants or wild type (4287). Error bars indicate standard deviation and capitals define statistically different groups (ANOVA, p = 0.95). (0.03 MB PDF) [file ppat.1000637.s011.pdf]

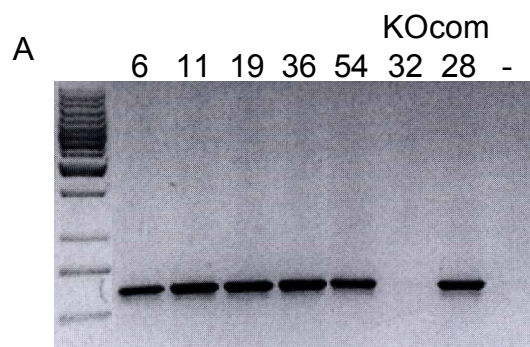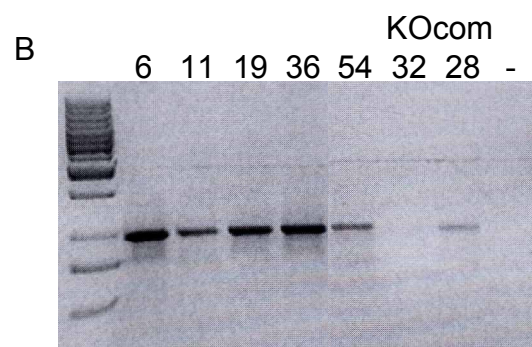

Supplement: Figure S11 — Analysis of transformants complemented with SGE1PM. A complementation construct containing a phleomycin expression cassette and the SGE1PM gene, encoding Sge1R66S, including 901 bp up- and 341 bp downstream sequences, was introduced in the SGE1 knock-out mutant #32 by Agrobacterium-mediated transformation. A) Verification of the presence of the SGE1 ORF by PCR using primers c and d (see Figure S1A). B) Verification of homologous recombination by PCR using primers a and b (see Figure S2). The 1 kb DNA ladder of Fermentas (www.fermentas.com) is used as a marker. −, negative control. Deletion mutant SGE1KO32 and complementation mutant SGE1com28 were used as a negative and positive control, respectively. (0.34 MB PDF) [file ppat.1000637.s012.pdf]
